# High-yield visible-to-ultraviolet upconversion by ZnSe quantum dots with dynamic triplet transfer

**DOI:** 10.1038/s41467-026-75613-5

**Published:** 2026-07-17

**Authors:** Xin Zhang, Rongxin Zhang, Lei Wang, Feng Chen, Shan He, Zihao Xu, Zhigang Xia, Guijie Liang

**Affiliations:** 1 Hubei Key Laboratory of Low Dimensional Optoelectronic Materials and Devices, Hubei University of Arts and Science, Xiangyang, China; 2 Department of Chemistry and the Hong Kong Branch of the Chinese National Engineering Research Center for Tissue Restoration and Reconstruction, The Hong Kong University of Science and Technology, Kowloon, China; 3 Huairou Research Center, Institute of Chemistry, Chinese Academy of Sciences, Beijing, China; 4 State Key Laboratory of New Textile Materials and Advanced Processing, Wuhan Textile University, Wuhan, China

**Keywords:** Photochemistry, Quantum dots, Quantum dots, Energy transfer

## Abstract

Triplet–triplet annihilation upconversion sensitized by quantum dot is often limited by the short exciton lifetime, necessitating tethered triplet transmitter. Here, we report a transmitter-free system of ZnSe quantum dot and 2,5-diphenyloxazole achieving 24% upconversion quantum yield, the highest for quantum dot sensitization upconversion. The upconversion quantum yield peaks at 30 mM 2,5-diphenyloxazole. Transient spectroscopy shows the triplet energy transfer efficiency is over 80% regardless of the 2,5-diphenyloxazole concentration, but the triplet–triplet annihilation efficiency dominates the overall upconversion quantum yield and shows a strong dependence on the 2,5-diphenyloxazole concentration that increases first from 10% to 25%, then decreases. The optimal concentration is achieved when the triplet 2,5-diphenyloxazole collision probability is highest, thus ensuring a maximum triplet–triplet annihilation efficiency. This work demonstrates a design principle of binary quantum dot upconversion system and reveals the critical role of modulating emitter concentration for optimal upconversion quantum yield.

## Abstract (web summary)

Triplet–triplet annihilation upconversion sensitized by quantum dots generally requires triplet transmitters tethered on the quantum dot. This work reports a transmitter-free system with 24% quantum yield, the highest for QD sensitizers so far.

## Introduction

Triplet–triplet annihilation photon upconversion (TTA-UC) represents an attractive wavelength conversion strategy, in contrast to nonlinear optical processes, can operate efficiently under solar irradiation^1^. By converting the abundant near-infrared photons in sunlight into visible photons, TTA-UC has the potential to improve the efficiency of photovoltaic devices, surpassing the Shockley–Queisser limit^2–5^. TTA-UC has been considered a promising approach in solar energy harvesting. Beyond photovoltaics, TTA-UC recently gained attention in sensitizing photochemical transformations^6–12^, where the high-energy singlet states of annihilators can transfer charge or energy to substrate molecules, thus initiating chemical reactions.

**Fig. 1:**
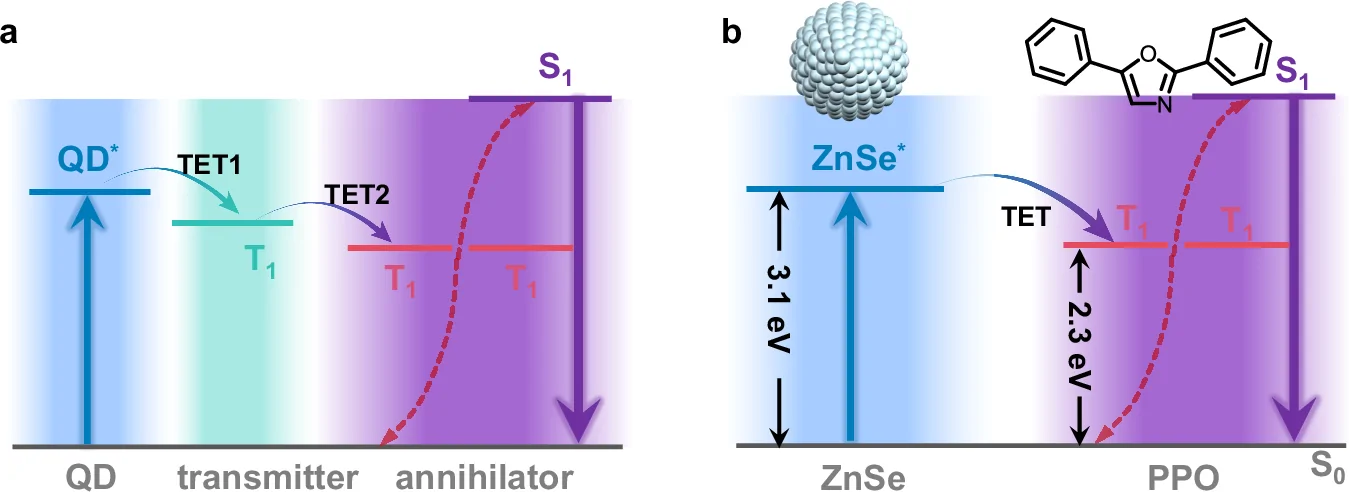
Mechanism comparison between QD-sensitized photon upconversion systems with and without transmitter. **a** Schematic illustration of a conventional QD-sensitized photon upconversion system consisted of a QD photosensitizer, a triplet transmitter and an annihilator. Excitation energy is transferred from the QD to the annihilator triplet state via a two-step triplet energy transfer (TET) process. **b** Transmitter-free ZnSe QD-sensitized visible-to-UV upconversion system developed in this work, consisting ZnSe QDs as the photosensitizer and PPO as the annihilator. In this configuration, the ZnSe exciton (3.1 eV) directly transfers energy to the PPO triplet state (2.3 eV) without intermediate acceptors. Note: ZnSe exciton energy is calculated from the PL peak, PPO triplet energy is obtained from previous literature (Supplementary Fig. 1 shows accurate energy levels). S_0_, S_1_ and T_1_ represent the ground state, singlet excited state and triplet excited state, respectively, for corresponding components in the TTA-UC system.

In a typical TTA-UC system, the sensitizer absorbs low-energy photons and transfers the energy to an annihilator via triplet energy transfer (TET). Because such transfer often relies on diffusion-collision mechanisms in solution, a long triplet lifetime of the sensitizer is essential. Consequently, common molecular sensitizers are organometallic complexes^13–16^ and thermally activated delayed fluorescence dyes^9,17,18^, both of which efficiently harvest photon energy and populate long-lived triplet states. Colloidal quantum dots (QDs) have also emerged as attractive sensitizers due to their size-tunable absorption/emission wavelengths^19^, high photostability^20,21^, and large extinction coefficients^22^. However, the exciton lifetime of QDs is typically limited to the nanosecond range^23–25^, which is far shorter than microsecond range of molecular triplets. To address this limitation, conventional QD-sensitized TTA-UC strategies employ triplet transmitter molecules with specific anchoring groups covalently bound to the QD surface^7,12,26–39^. In these systems, the QD exciton energy is first transferred to the triplet transmitter via TET, and subsequently relayed to the annihilator triplet, ultimately producing upconverted emission (Fig. 1). While feasible, this two-step TET process inherently limits both TET efficiency and UC quantum yield (UCQY), and the need for chemically modified triplet transmitter increases the complexity of system design.

Herein, we report a strategy that directly sensitizes 2,5-diphenyloxazole (PPO) with ZnSe QDs, eliminating the need for intermediate triplet transmitter. This direct TET from the QD exciton to the PPO triplet state achieves an efficiency of ~93.1%, enabling a visible-to-ultraviolet (vis-to-UV) UCQY as high as 24% (Fig. 1). It is worth pointing out that this UCQY is the highest in QD system^29,31,37,40–42^, which demonstrates this design reaches a balance of simplicity and efficiency. Leveraging this high UCQY, the ZnSe PPO system demonstrates excellent performance in sub bandgap photocatalytic polymerization reactions, highlighting its potential for efficient visible-to-UV photon upconversion under mild conditions.

## Results

**Fig. 2:**
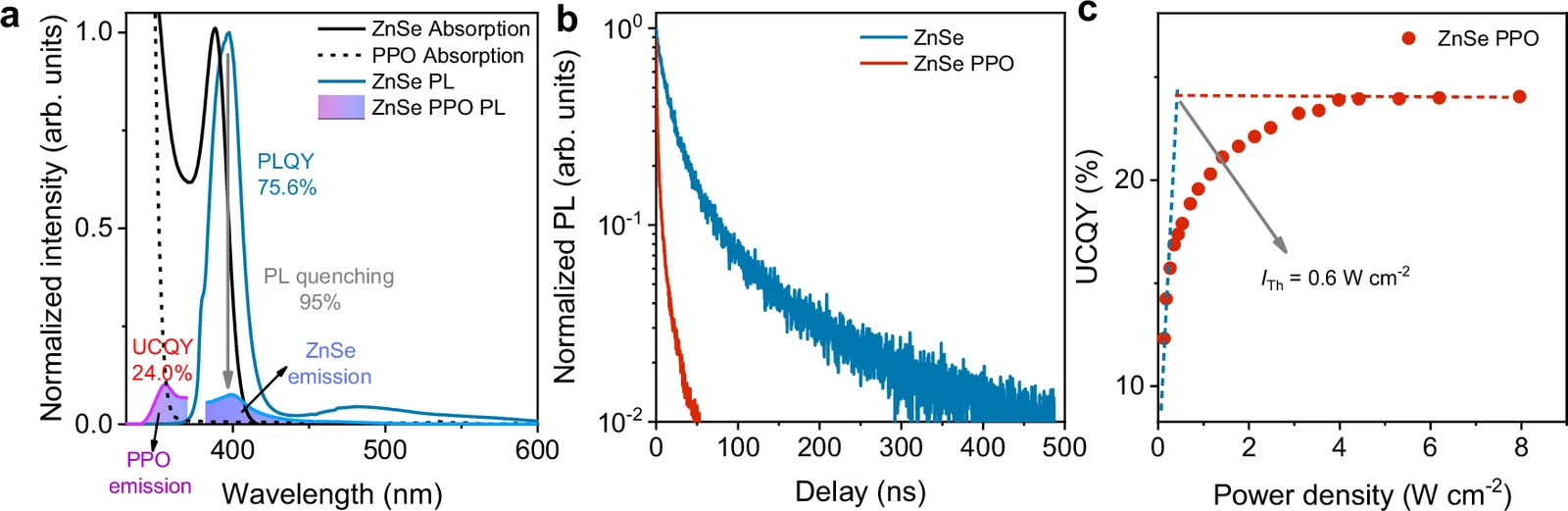
Photon upconversion of ZnSe PPO. **a** Normalized absorption and PL spectra of ZnSe QD, PPO and ZnSe PPO under 380 nm excitation. The ZnSe PPO PL is separated into contributions from ZnSe QD and PPO PL. **b** Time-resolved PL kinetics of ZnSe (blue) and ZnSe PPO (red) probed at 397 nm. **c** Calculated TTA-UC efficiencies at varying excitation power densities. Note: the wavelength of excitation laser is 380 nm. Source data for (**a–c**) are provided as a Source Data file.

Colloidal ZnSe quantum dots (QDs) were synthesized following previously reported procedures^24,25^. The ZnSe QD first excitonic absorption peak is at 387 nm and corresponding photoluminescence (PL) peak is at 397 nm (Fig. 2a). The QDs exhibited a Stokes shift of ~10 nm. Transmission electron microscopy (TEM) showed the particle size is 2.9 ± 0.9 nm (Supplementary Fig. 2). The correlation between the first excitonic peak position and the particle size is consistent with previous studies^22^. The photoluminescence quantum yield (PLQY) is measured to be 75.6%. The QD also displayed a pronounced trap-state emission at ~480 nm, which could trap excitons and lower PLQY (Fig. 2a). Upon addition of PPO to ZnSe, the QD excitonic emission was quenched by ~95%. The PL quenching of ZnSe QD is accompanied by the emergence of PPO singlet emission at ~355 nm. The upconversion quantum yield (UCQY), determined using a relative method, reached ~24% (Fig. 2a and Methods). Time-resolved photoluminescence of ZnSe QD in Fig. 2b showed much faster decay with PPO, also indicating efficient TET from ZnSe QD to PPO. Power-dependent UCQY measurements further confirmed the TTA-UC mechanism (Fig. 2c). At low excitation power densities, UCQY increased with increasing excitation power density, plateauing beyond an excitation power threshold that is characteristic for TTA-UC. The threshold for ZnSe PPO is determined to be 0.6 W cm^−2^. ZnSe PPO demonstrated the highest UCQY of 24% reported, establishing it as the most efficient TTA-UC system. Considering the 380 nm excitation lies near the UV region, we further investigated TTA-UC performance of the ZnSe PPO hybrids under 405 nm excitation (Supplementary Fig. 3). As it shows, UCQY under 405 nm excitation is 16.9%, lower than that under 380 nm excitation but the anti-Stokes shift is increased from ~230 meV to ~431 meV. Considering the inhomogeneity of ZnSe QD (2.9 ± 0.9 nm), this reduction of anti-Stokes shift can be attributed to the increased QD size, which leads to a lower degree of exciton wavefunction delocalization beyond the QD surface, thereby decreasing the electronic coupling with molecular orbitals. In addition, the reduced energetic driving force further limits the efficiency of the Dexter-type TET process. Control experiment of direct exciting PPO at 30 mM under 380 nm excitation shows no emission at 350 nm, thus excluding the emission from thermally assisted hot-band absorption^43^. As Supplementary Fig. 4 shows, under both 1.63 and 5.85 W cm^−2^ excitation power densities, no emission was detectable around 355 nm.

**Fig. 3:**
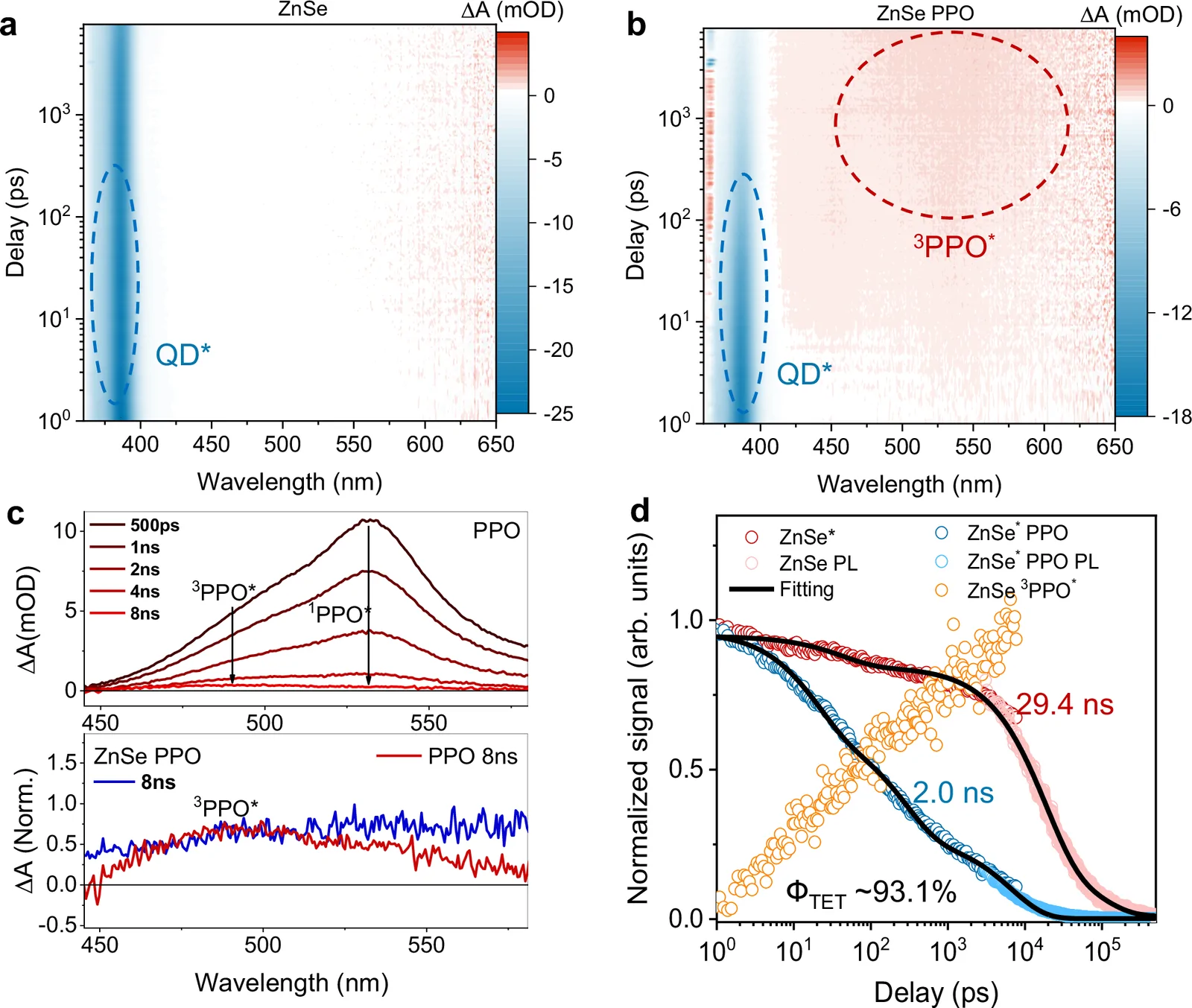
Energy transfer from ZnSe QD exciton state to PPO triplet state. Two-dimensional pseudo-color TA spectra of (**a**) ZnSe and **b** ZnSe PPO following excitation by a 370 nm pump. QD^*^ and ^3^PPO^*^ represent the singlet excited state of QD and the triplet excited state of PPO, respectively. **c** TA spectra of PPO at indicated delay times following excitation by a 300 nm pump. TA spectra of the ZnSe PPO at 8 ns is also plotted for comparison. **d** Normalized exciton bleach (XB) kinetics of ZnSe (red) and ZnSe PPO (blue) probed at 397 nm. Time-resolved PL kinetics of ZnSe (light red) and ZnSe PPO (light blue), normalized to the XB signal at ~3 ns, are overlaid to illustrate exciton kinetics. ^3^PPO^*^ kinetics (yellow) were extracted by averaging the TA signals of ZnSe PPO over the 450-600 nm range. Note: the concentration of PPO is 30 mM. Source data for (**a–d**) are provided as a Source Data file.

The triplet energy transfer from photoexcited QD to PPO is studied by transient absorption (TA) spectroscopy. The main TA feature of pristine ZnSe QD after 370 nm excitation is the exciton bleach (XB) centered at 397 nm, reflecting the excitonic (QD^*^) kinetics (Fig. 3a). Upon addition of PPO, the XB decay rate drastically increased, accompanied by the emergence of a new excited-state absorption (ESA) band in the 450–600 nm region (Fig. 3b). To assign this signal, PPO was directly photoexcited by 300 nm pump. An ESA peak at 530 nm corresponding to the S_1_–S_n_ transition of PPO singlet was observed, followed by the development (after ~1 ns) of a broad 450–600 nm ESA band, characteristic of the ^3^PPO^*^ generated via intersystem crossing (ISC) (Fig. 3c), in agreement with literature reports^24,41^. This confirms that the 450–600 nm feature in Fig. 3b arises from ^3^PPO^*^ formation concurrent with QD^*^ decay.

Kinetic traces of the XB at 397 nm and the ^3^PPO^*^ ESA signal at 450–600 nm are shown in Fig. 3d. Their kinetics show similar time constant, which directly evidences TET from ZnSe^*^ to ^3^PPO^*^. Combining transient PL and TA data, a three-exponential fit (Methods and Supplementary Table 1) yielded a ZnSe^*^ lifetime decrease from 29.4 to 2.0 ns upon PPO addition, corresponding to a TET efficiency of 93.1%. The XB quenching efficiency determined by TA is consistent with the steady-state PL quenching efficiency (95%). These results confirm that exciton PL quenching is primarily due to TET rather than charge transfer. Notably, this single-step TET process achieves higher efficiency (93.1%) and UCQY (24%) than the two-step TET in CsPbBr_3_–phenanthrene-PPO systems (88% and 12.2%, respectively)^42^, highlighting the advantage of a simplified architecture for enhancing both TET and UC performance.

We also note that the emission from the ZnSe trap state is strongly quenched upon addition of PPO. It suggests that trap states could participate in TET, for example via transfer to ^3^PPO^*^. However, steady-state PL, transient spectroscopy, and quantitative efficiency analysis indicate that trap-mediated transfer is unlikely to dominate the observed high UCQY. Firstly, the PL of ZnSe is primarily governed by band-edge exciton emission at 397 nm, while trap emission represents only a minor fraction of the total PL intensity (PLQY ~ 8.3%). Even assuming unity TET efficiency from trap states, their limited population would be insufficient to explain the observed UCQY of 24%. Secondly, addition of PPO leads to strong quenching of the band-edge exciton emission (~95%), indicating efficient electronic coupling between QDs and PPO. TA measurements further confirm efficient TET, evidenced by accelerated decay of the exciton signal together with the emergence of characteristic ^3^PPO^*^ absorption features. These results clearly support direct TET from the band-edge exciton as the dominant pathway.

**Fig. 4:**
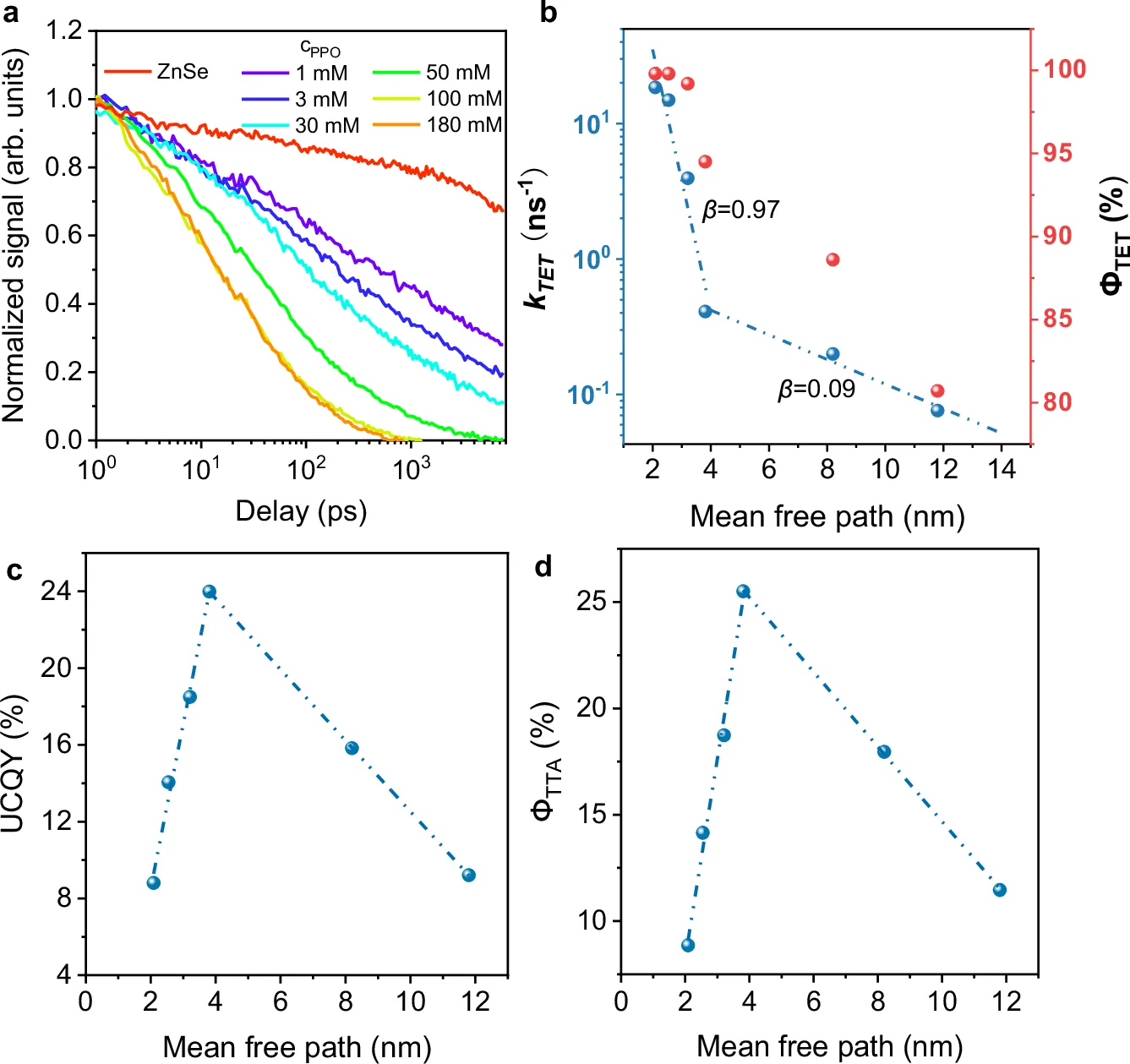
TET rate constants, UCQYs and TTA efficiencies at different mean free paths of PPO. **a** Normalized exciton bleach (XB) kinetics of ZnSe and ZnSe with varying PPO concentrations probed at 397 nm. **b** Triplet energy transfer rate constants (*k*_TET_) (blue symbols) and calculated transfer efficiency (*Φ*_TET_) (red symbols). **c** Measured UCQY at different mean free path of PPO. **d** Calculated TTA efficiency at different mean free path. Source data for (**a–d**) are provided as a Source Data file.

The XB decay kinetics of ZnSe at varying PPO concentrations were measured for the dependence of TET rate. As PPO concentration increased, the XB decay rate accelerated (Fig. 4a). Here, the fitted *k*_TET_ is plotted against the mean free path (Methods) of the PPO molecule. The *k*_TET_ is logarithmic linear to the mean free path (*k*_TET_∝e^-*βr*^), which is consistent with the Dexter energy transfer theory^44,45^. With PPO concentration increases, the slope *β* showed a sharp increase from 0.09 to 0.97 with a turn point at 3.8 nm mean free path (Fig. 4b). The increased slope (0.97) below 3.8 nm indicates the TET between QD and PPO follows a different mechanism than above 3.8 nm, possibly due to a difference between direct TET and sequential TET, where the latter requires the PPO to diffuse to QD first. Notably, this distance of 3.8 nm matches the mean diameter of ZnSe, implying that under such conditions, at least one PPO molecule is located within the immediate vicinity of a ZnSe QD. In this regime, TET can occur without relying on diffusion, explaining why high TET efficiency is achieved even without PPO anchoring. The TET efficiency, due to the fast TET rate, are above 80% for all the PPO concentrations we studied as shown in Fig. 4b.

UCQY at varying PPO concentrations shows an optimal performance at 30 mM PPO, corresponding to a mean free path between PPO molecule of ~3.8 nm (Fig. 4c). While further increasing PPO concentration shortens the mean free path and increases *k*_TET_, UCQY starts to decrease. Considering the overall UCQY is the product of *Φ*_TET_ and *Φ*_TTA_, we used the measured UCQY and *Φ*_TET_ to calculate the *Φ*_TTA_. As shown in Fig. 4d, the *Φ*_TTA_ also shows a similar trend to UCQY in regarding to PPO concentration that peaks at 3.8 nm mean free path. It is worth pointing out since the *Φ*_TET_ is fairly independent on the PPO concentration, the UCQY is mostly dictated by *Φ*_TTA_. This can be explained by that at excessively high PPO concentrations, the probability of an individual ^3^PPO^*^ encountering another ^3^PPO^*^ decreases due to the molecular crowding^46,47^. Thus, the maximum UCQY is obtained at ~30 mM PPO, where the balance between efficient TET and optimal TTA is achieved.

Previous reports have shown that, due to the short lifetimes of QD excitons (ns scale), efficient TET between QDs and molecules typically requires anchoring the molecules onto the QD surface via specific ligands^7,12,26–39^. In the reference^48^, it has also been reported that the weak interactions between sulfur atoms in annihilators and PbS enabling dynamic attachment and detachment promote triplet exciton transfer. However, unlike the measurable blue shift of the QD excitonic absorption peak in the reference^48^, no measurable shift in the excitonic absorption peak of ZnSe QDs is observed upon addition of PPO (Supplementary Fig. 5). The ^1^H NMR shows that the PPO molecules remain predominantly in a freely diffusing state rather than being coordinated to the QD surface (Supplementary Fig. 6). The chemical shifts and peak widths of both PPO and OA remaining essentially unchanged upon mixing, indicated that PPO exists in the free molecular state in solution rather than being surface bound. This suggests that TET from ZnSe^*^ to ^3^PPO^*^ likely proceeds via diffusion-controlled collisions. The high TET efficiency obtained from TA measurement suggests that the free PPO can accept the triplet energy from QD very efficiently, which is less reported^49^. By controlling the molecular distance, we believe that the transformation from a tunneling mechanism to a dynamic mechanism occurs with the mean free path increasing over 3.8 nm. The highest UCQY is obtained exactly at the turning point. Specifically, TA analysis on *k*_TET_ in Fig. 4 reveals much larger *β* (0.97 nm^-1^) below 3.8 nm mean free path, which can be attributed to the tunneling mechanism that TET can occur without relying on diffusion. On the contrary, when the mean free path exceeds 3.8 nm, the much smaller *β* (0.09 nm^-1^) indicates a similar dynamic mechanism where the acceptor diffuses close to the QD^49^. Consequently, at high PPO concentrations exceeding 30 mM, the tunneling mechanism dominates the TET process, while the dynamic mechanism dominates at low PPO concentrations.

**Fig. 5:**
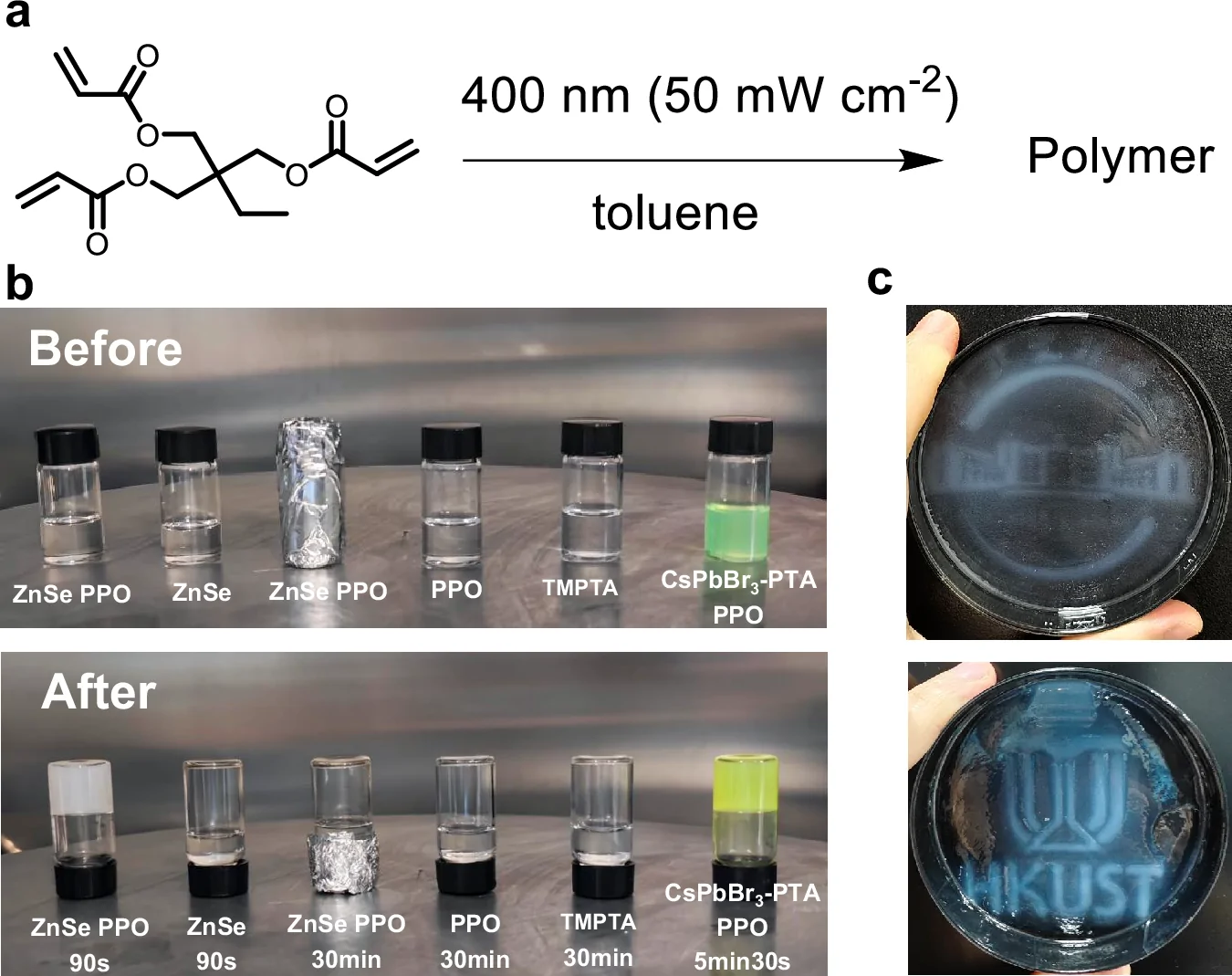
Photopolymerization performance and 3D printing demonstration enabled by ZnSe PPO sensitization. **a** Schematic illustration of TMPTA photopolymerization. **b** Photopolymerization performance of various sensitizer systems under 400 nm irradiation. **c** Demonstration of 3D printing enabled by efficient photopolymerization using the ZnSe PPO sensitizer.

Leveraging the high UCQY of ZnSe PPO (24%), we employed this binary system to sensitize the photopolymerization of trimethylolpropane triacrylate (TMPTA) at sub band gap irradiation (Fig. 5a). Given the strong reducing power of PPO singlets, no additional initiators or sacrificial agents are required. Under 400 nm irradiation at 50 mW cm^-2^, complete polymerization of ZnSe PPO TMPTA was achieved within 90 seconds (Fig. 5b). In contrast, ZnSe TMPTA, PPO TMPTA, pure TMPTA, and ZnSe PPO TMPTA kept in the dark (wrapped in aluminum foil) showed no polymerization after 90 s, confirming that TTA-UC is the key process for the reaction. For comparison, a CsPbBr_3_-PTA PPO TMPTA system with 12.2% UCQY required 330 s to reach complete polymerization (Fig. 5b), 3.6 times longer. Notably, the UCQY of ZnSe PPO is two times higher than that of CsPbBr_3_–PTA PPO, its polymerization rate is enhanced by a factor of 3.6, highlighting the superior performance of a simple, ligand-free system in photopolymerization. We further exploited the high polymerization efficiency of ZnSe PPO TMPTA in a proof-of-concept 3D printing demonstration (Fig. 5c). Under 400 nm irradiation for 10 min, the ZnSe PPO TMPTA mixture, poured into a Petri dish and covered with a photomask of the desired pattern, produced the patterned polymer structures.

In summary, we have demonstrated a transmitter-free ZnSe QD PPO hybrid system that achieves a high UCQY of 24% via a one-step TET pathway with ~93.1% efficiency. TA measurements reveal that a mean free path between PPO molecules of ~3.8 nm, comparable to the ZnSe QD diameter, enables near-unity TET between ZnSe QD and PPO and optimal TTA efficiency between PPO triplets, circumventing the diffusion limitation typically observed in nanosecond-lifetime QDs. Compared to the multistep CsPbBr_3_–PTA PPO platform, our minimalist design not only improves UCQY but also enhances photopolymerization performance by a factor of 3.6, enabling initiator-free 3D printing. These findings establish a generalizable strategy for designing high-efficiency, tether-free TTA-UC systems and broaden their potential in photochemical manufacturing and related photonic applications.

## Methods

### Chemicals

Anhydrous zinc acetate (Zn(Ac)_2_, Alfa, 99.98%), diphenylphosphine (DPP, Innochem, 98%), sublimed sulfur (S, Innochem, 99.9%), zinc stearate (ZnSt_2_, Sigma-Aldrich), oleic acid (OA, Sigma-Aldrich, 90%), oleylamine (OAM, Sigma-Aldrich, 70%), 1-octadecene (ODE, Sigma-Aldrich, 90%), acetone (Acros, >99%), trioctylphosphine (TOP, Sigma-Aldrich, 97%), selenium powder (Se, Sigma-aldrich, 99.9%), ethanol (Innochem, 97%), n-hexane (Innochem, 99%), ethyl acetate (Innochem, 99%), 2,5-diphenyloxazole (Tokyo chemical industry corporation, 99%), trimethylolpropane triacrylate (Innochem, 85%).

### QD synthesis

The synthesis method refers to literature with some modifications^24,25^. A mixture of Zn(Ac)_2_ (0.4588 g), OA (3 mL), OAM (1 mL), and ODE (15 mL) was degassed at 120 °C for 60 min under vacuum. The solution was then heated to 303 °C under N_2_ flow. A selenium precursor solution (0.6 mL of Se-DPP, 0.3147 g Se powder in 2 mL DPP) was then injected, and the reaction was allowed to proceed for 7 min, to yield ZnSe QDs. The resulting QDs were purified by two centrifugation cycles (6800 g, 5 min each), first to remove unreacted salts and then to precipitate the product using an anti-solvent mixture (14 mL, ethanol: ethyl acetate = 1:1). The final QD products were dispersed in 5 mL hexane.

### TEM measurements

TEM images were acquired on a HITACHI H-7650 TEM at 100 KV (line resolution of 0.204 nm) by drop casting the QD-hexane dispersions on carbon grids and drying under ambient conditions.

### Molecular structure characterizations

Nuclear magnetic resonance (NMR) spectra were measured on a Bruker AVANCE NEO 600 MHz NMR spectrometer.

### Transient absorption

The pump-probe transient absorption spectroscopy setup is consisted of a commercial Ti: Sapphire laser and a commercial transient absorption setup (Helios, Ultrafast Systems). The setup utilized an 800 nm output pulse from a 1 kHz regenerative amplifier, which was split into two beams by a 50% beam splitter. One beam is used as the pump, directed to a TOPAS optical parametric amplifier (OPA) to generate a tunable excitation pulse. The other beam was used to generate the probe. A small portion (<10%) of this beam was attenuated by a neutral density filter and focused into a nonlinear crystal to produce a white light continuum (WLC) as probe. The time delay between the pump and probe pulses was controlled by a motorized delay stage. The transient absorption data was recorded by Ultrafast Systems Helios software.

### Photopolymerization and 3D printing procedures

QDs were dispersed in toluene and diluted to achieve an absorbance of 1.0 at 400 nm. 1.0 mL of this QD solution, 0.2 mL of PPO (0.2 M in toluene) and 1.2 mL of TMPTA were added and mixed thoroughly. The mixture was irradiated with a 400 nm light source for varying durations to evaluate photopolymerization efficiency. For the 3D printing demonstration, the above ZnSe PPO TMPTA mixture was poured into a Petri dish and covered with a photomask of the desired pattern. The sample was then irradiated with 400 nm light for 10 min to produce the patterned polymer structures.

### TTA-UC measurements

UCQY is defined as the ratio between the number of emitted upconverted photons and the number of absorbed excitation photons. Following the standard convention in the TTA-UC field^29^, the UCQY is expressed as Eq. (1):

1 $${{\Phi }}_{{{{\rm{UC}}}}}=2{{\Phi }}_{{{{\rm{C307}}}}}\frac{1-{10}^{-{{A}}_{{{{\rm{C}}}}307}}}{1-{10}^{-{{A}}_{S}}}\frac{{F}_{{{\rm{S}}}}}{{F}_{{{{\rm{C}}}}307}}\frac{{n}_{{{\rm{S}}}}^{2}}{{n}_{{{{\rm{C}}}}307}^{2}}$$

Where *A*_*i*_ is the absorption (optical density) at the excitation wavelength, *F*_*i*_ is the integrated photon number and *n*_*i*_ is the refractive index of the solvent. Subscripts C307 and S are coumarin 307 and sample, respectively. It should be noted that, since the upconversion PL of PPO overlaps to some degree with the emission of ZnSe QDs, *F*_s_ is calculated by normalizing the pure PPO PL to the corresponding characteristic peak for the PL of QDs PPO hybrids.

The spectral response function of the detection system was calibrated using a photon-number-based method following a previously reported procedure^29^. Specifically, a femtosecond optical parametric amplifier (fs-OPA) was used to generate monochromatic excitation at different wavelengths. By measuring the laser power at each wavelength, the corresponding photon flux was calculated, allowing the construction of a calibration curve correlating detector signal intensity with absolute photon number.

To verify the accuracy of the calibration curve, two well-established fluorescent dyes, PPO and anthracene, were employed as cross-calibration standards. Based on literature values, PPO exhibits a fluorescence quantum yield of approximately 85% in cyclohexane^50^. Using the calibrated spectral response function, we determined the fluorescence quantum yield of anthracene to be 26% (Supplementary Fig. 7), which agrees well with widely accepted literature values^51^. This consistency confirms the reliability of the instrument calibration.

### Kinetics fitting model

All the kinetic curves of QDs and QDs PPO were fitted by a 3-exponential function. The average lifetime ${\tau }_{{ave}}$ was calculated by Eq. (2):

2 $${\tau }_{{{{\rm{ave}}}}}=\frac{{a}_{1}{\tau }_{1}+{a}_{2}{\tau }_{2}+{a}_{3}{\tau }_{3}}{{a}_{1}+{a}_{2}+{a}_{3}}$$

Where *τ*_i_ and *a*_i_ are the lifetime and amplitude of the *i*-th exponential component, respectively. The *k*_TET_ is calculated by $\frac{1}{{\tau }_{{{{\rm{QDPPO}}}}}}-\frac{1}{{\tau }_{{{{\rm{QD}}}}}}$. All the calculation results were listed in Supplementary Table 1 and Table 2.

### PPO mean free path calculation

The method for calculating the average free path of PPO molecules at different concentrations is as follows: assuming that PPO molecules are arranged in a cubic lattice in the solvent, at a concentration of *c* M, the corresponding lattice parameter for a single molecule can be calculated by Eq. (3):

3 $${{{\rm{mean}}}}\,{{{\rm{free}}}}\,{{{\rm{path}}}}={\left(\frac{1}{c\times 1000\times {{N}}_{{{\rm{a}}}}}\right)}^{\frac{1}{3}}\times {10}^{9}\,{{{\rm{nm}}}}$$

where *N*_a_ is Avogadro constant, the factor of 1000 converts liters to cubic meters, and the multiplier 10^9^ converts meters to nanometers.

## Supplementary information

Supplementary Information

Transparent Peer Review file

## Source data

Source Data

## Data Availability

All data are available in the main text or the supplementary information. Source data are provided with this paper.
