# Supplementary material for: High-yield visible-to-ultraviolet upconversion by ZnSe quantum dots with dynamic triplet transfer
Source: Nat Commun. 2026 Jul 17;17:8777. doi: 10.1038/s41467-026-75613-5 (PMC13493803; doi:10.1038/s41467-026-75613-5)
Supplement: Supplementary file 1 [file 41467_2026_75613_MOESM1_ESM.pdf]

Supplementary Information

High Yield Visible-to-Ultraviolet Upconversion by ZnSe Quantum Dots with Dynamic Triplet Transfer

Xin Zhang,[1,#*] Rongxin Zhang,[1#] Lei Wang,[1] Feng Chen,[1] Shan He,[2,*] Zihao Xu,[3,*] Zhigang Xia,[4] Guijie Liang,[1,*]

[1] Hubei Key Laboratory of Low Dimensional Optoelectronic Materials and Devices, Hubei University of Arts and Science, Xiangyang, Hubei 441053, China

[2] Department of Chemistry and the Hong Kong Branch of the Chinese National Engineering Research Center for Tissue Restoration and Reconstruction, The Hong Kong University of Science and Technology, Kowloon, Hong Kong 999077, China

[3] Huairou Research Center, Institute of Chemistry, Chinese Academy of Sciences, Beijing 100190, China

[4] State Key Laboratory of New Textile Materials and Advanced Processing, Wuhan Textile University, Wuhan 430200, China

[#] These authors contributed equally: Xin Zhang, Rongxin Zhang

[*]Address correspondence to: xinzhang@hbuas.edu.cn; shanhe@ust.hk; zihaoxu@iccas.ac.cn; guijie-liang@hbuas.edu.cn.

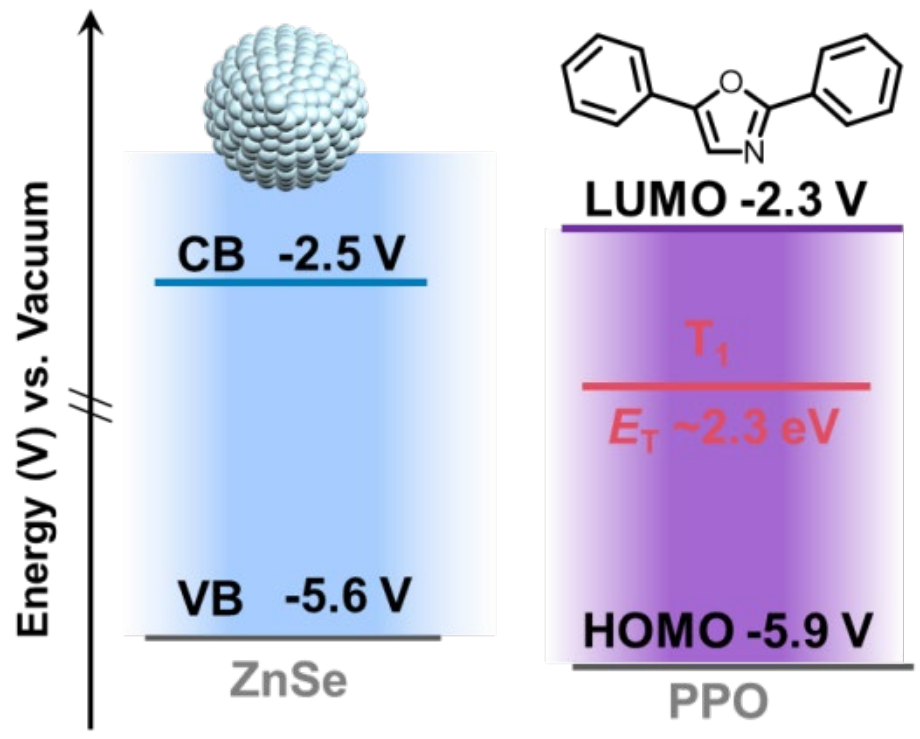

Supplementary Figure 1. Accurate energy levels. Conduction and valence band edge of ZnSe QD and HOMO, LUMO of PPO.[1-3]

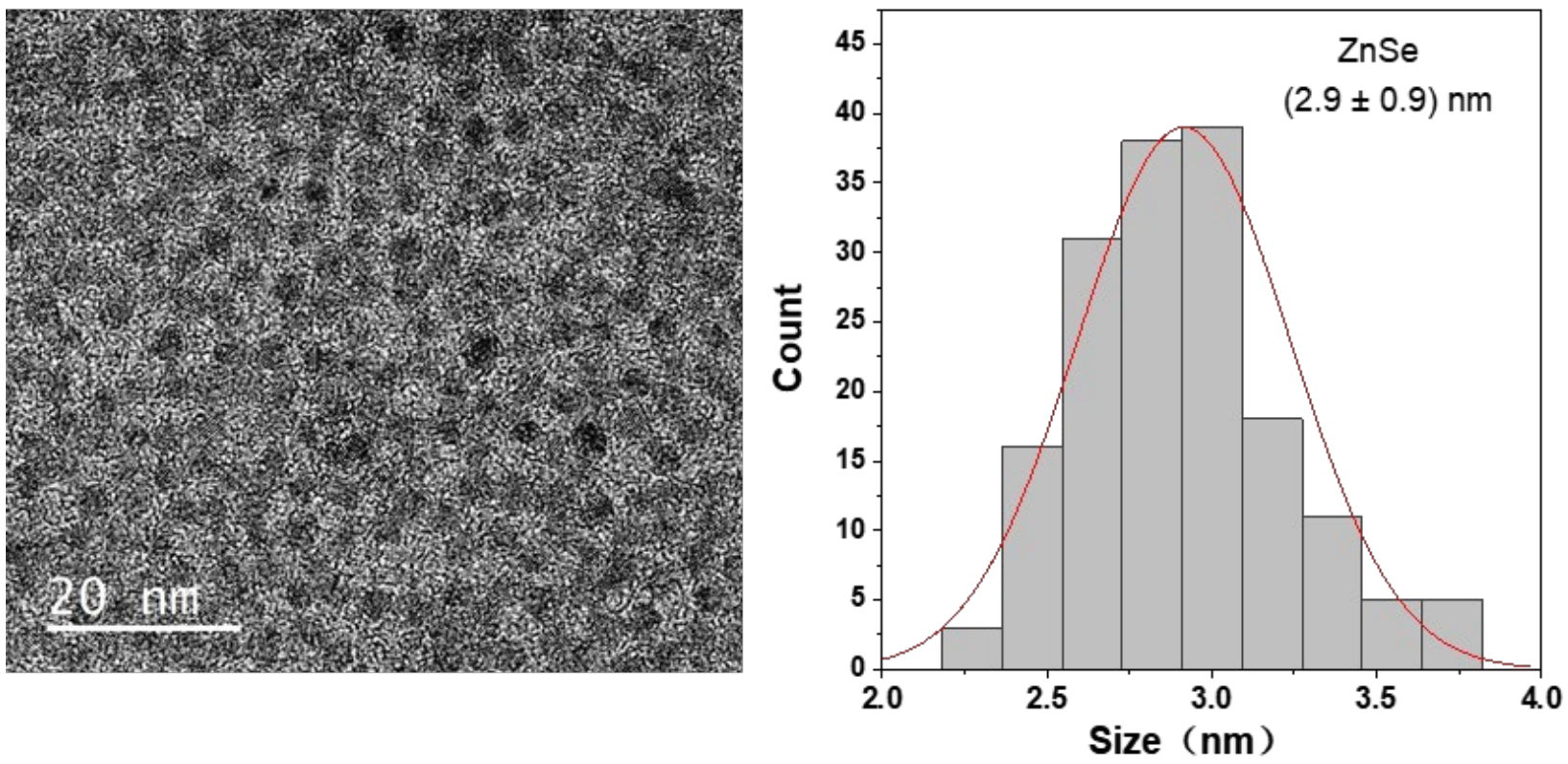

Supplementary Figure 2. TEM image. TEM image and size distribution of ZnSe QD. Source data are provided as a Source Data file.

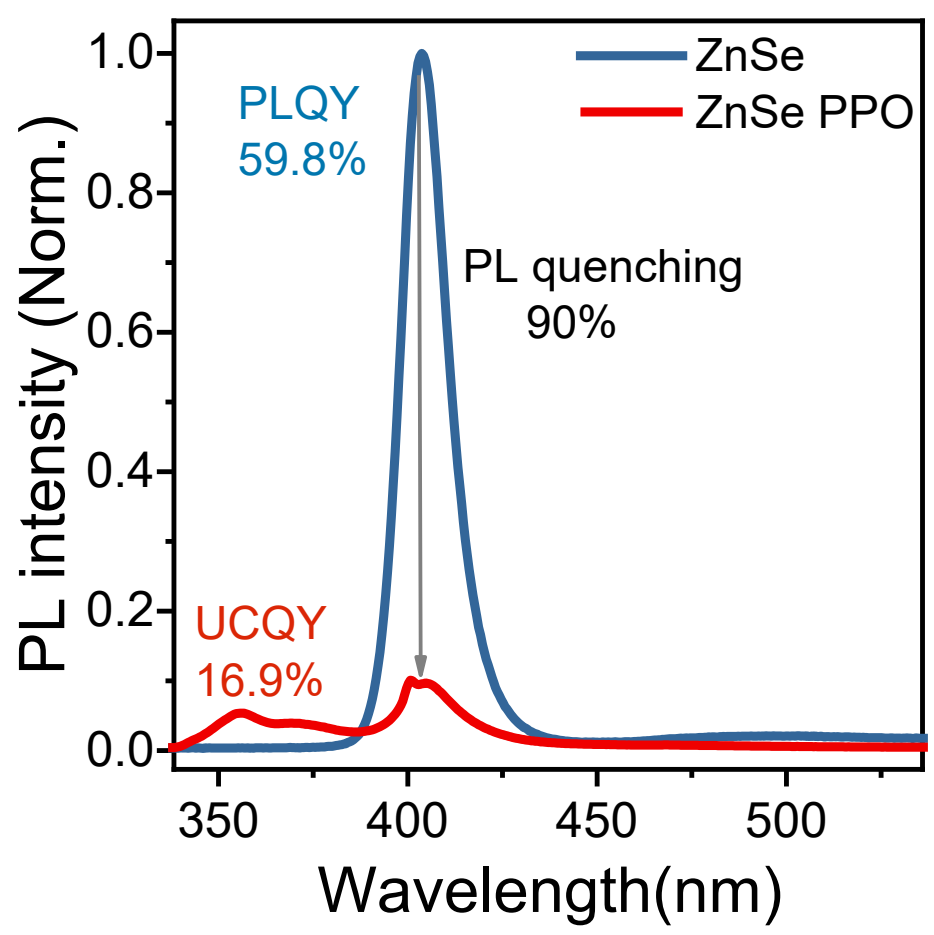

Supplementary Figure 3. ZnSe QD–sensitized visible-to-UV photon upconversion system under 405 nm excitation. PL spectra of ZnSe and ZnSe PPO under 405 nm excitation. Source data are provided as a Source Data file.

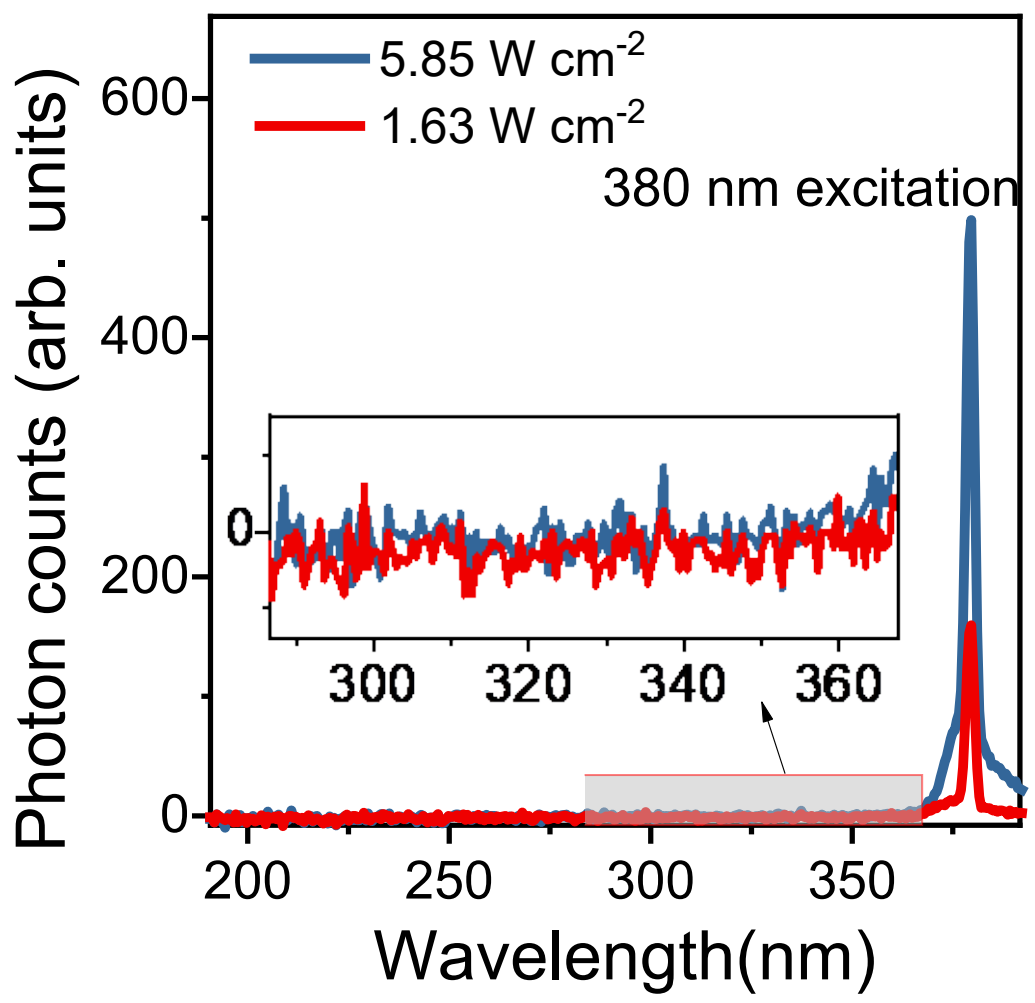

Supplementary Figure 4. Control experiment. PL spectra of 30 mM PPO under 380 nm excitation with 1.63 W cm^{-2} and 5.85 W cm^{-2} power densities. Source data are provided as a Source Data file.

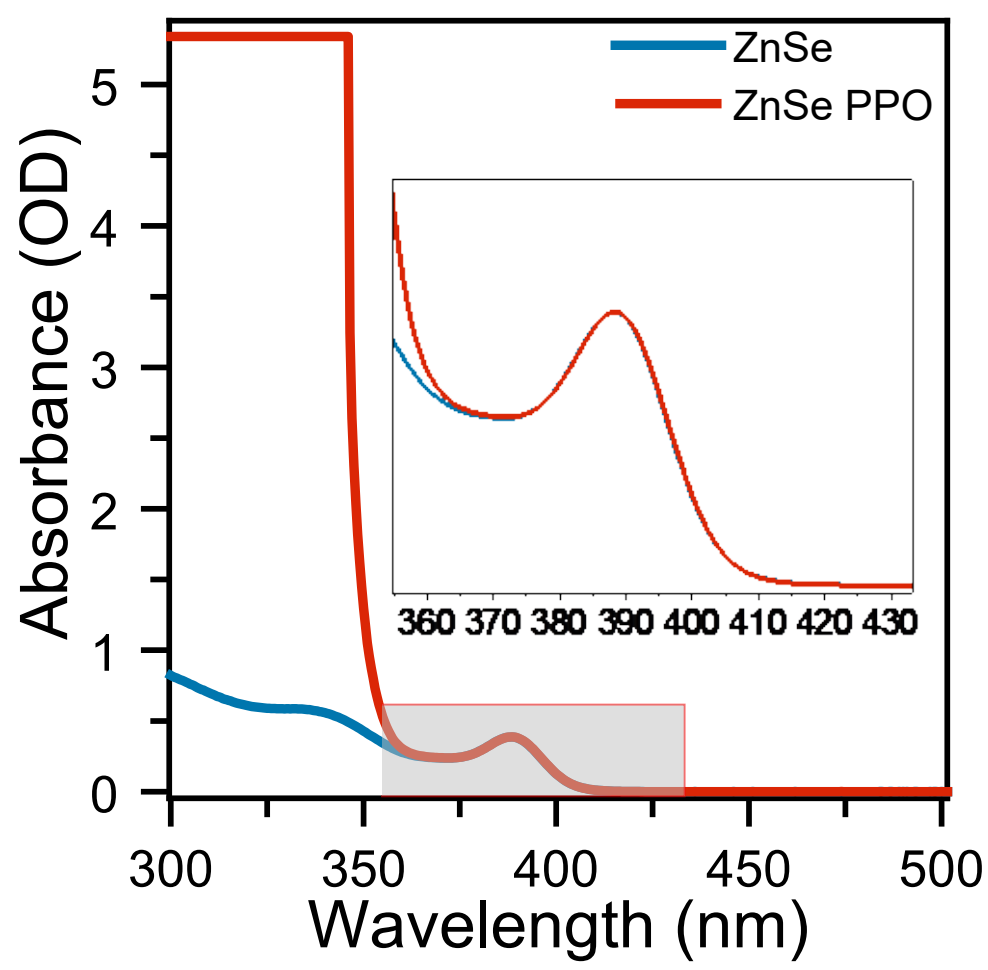

Supplementary Figure 5. Absorption spectra of QD and QD PPO. Absorption spectra of ZnSe (blue) and ZnSe PPO (red). The inset shows a magnified view of the region around the first exciton absorption peak. Source data are provided as a Source Data file.

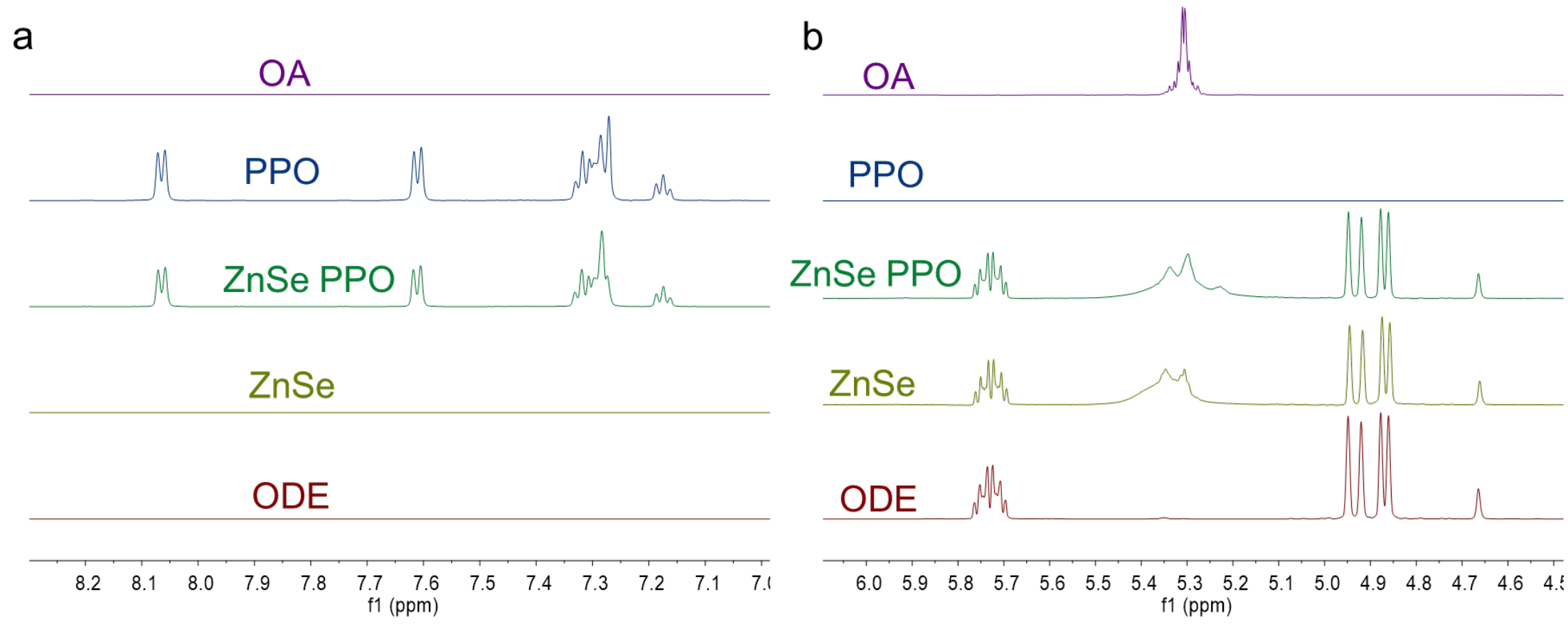

Supplementary Figure 6. ¹H NMR spectra. ¹H NMR spectra (in cyclohexane-d12) of OA (violet), PPO (blue) ZnSe PPO (green), ZnSe (yellow) and ODE (red). The PPO concentration is 30 mM in the PPO and ZnSe PPO within (a) 6.99-8.30 ppm and (b) 4.50-6.32 ppm. The QD concentration is 10 mg/mL in the ZnSe PPO and ZnSe. Source data for (a-b) are provided as a Source Data file.

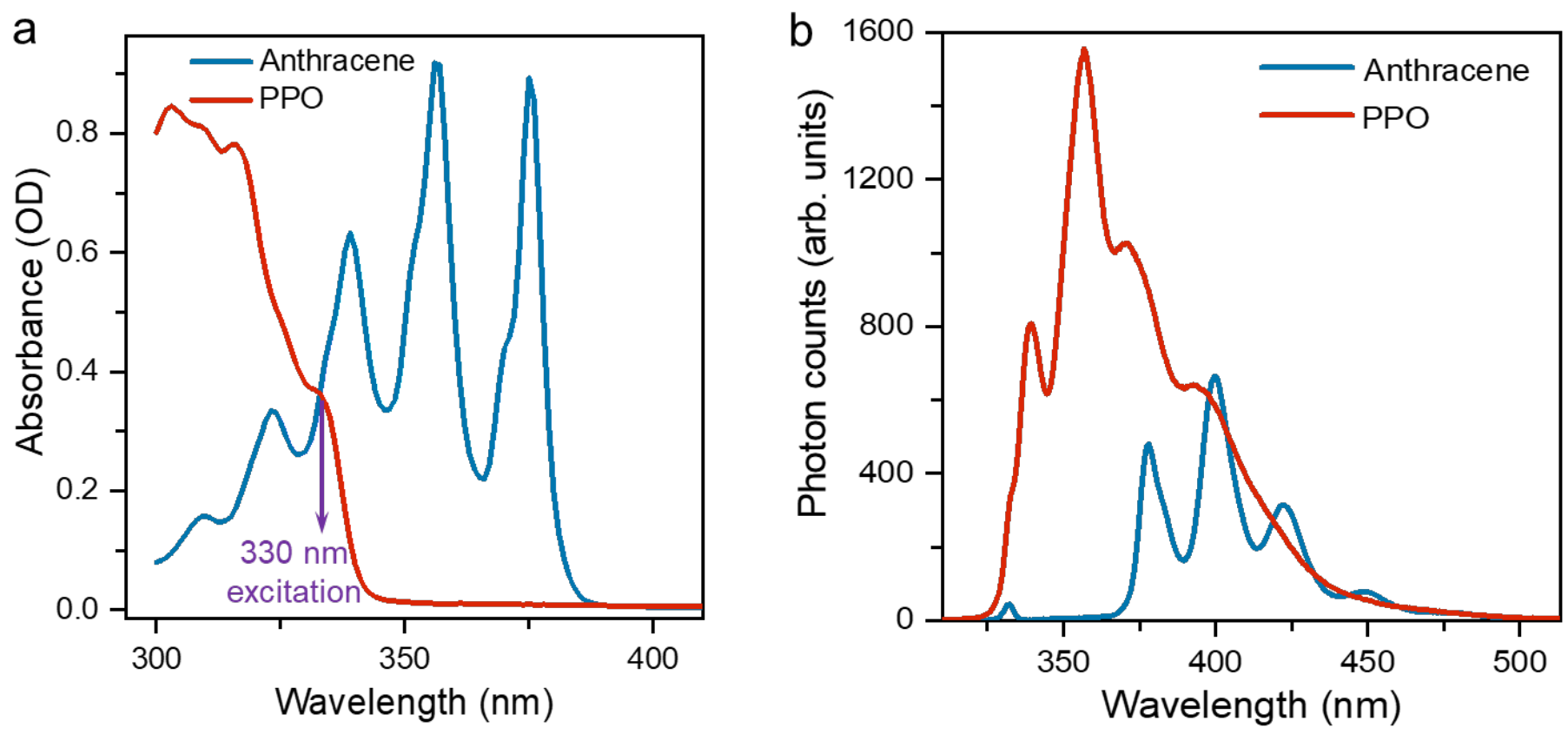

Supplementary Figure 7. Calibration of PLQY of anthracene using PPO (a) Absorption spectra of anthracene (blue) and PPO (red). Note: the absorbance at 330 nm for these two molecules is identical. (b) PL spectra of anthracene (blue) and PPO (red) under the 330 nm laser excitation. Note: the excitation source is the fs pulse produced by OPA. Source data for (a-b) are provided as a Source Data file.

Supplementary Table 1. Exciton lifetimes and k_{TET} of ZnSe and ZnSe PPO from TA and time-resolved PL spectra

	a_1	τ_1 (ps)	a_2	τ_2 (ps)	a_3	τ_3 (ps)	τ_{ave} (ns)	k_{TET} (ns^{-1})
$ZnSe^*$	0.65	17399	0.1	36	0.18	89247	29.4	
$ZnSe^*$ PPO	0.35	22	0.34	312	0.26	6945	2.0	0.461
ZnSe $^3PPO^*$	0.32	6.5	0.33	110.9	0.35	3209	1.2	

Supplementary Table 2. Exciton lifetimes and k_{TET} of ZnSe with varying PPO concentrations

c_{PPO} (mM)	mean free path (nm)	a_1	τ_1 (ps)	a_2	τ_2 (ps)	a_3	τ_3 (ps)	τ_{ave} (ps)	k_{TET} (ns^{-1})
180	2.1	0.3	3.73	0.43	26.6	0.26	156.6	53.8	18.5
100	2.6	0.29	3.08	0.47	29.4	0.22	230.1	66.7	15.0
50	3.2	0.3	5.94	0.46	67.6	0.22	952.1	247.3	4.0
30	3.8	0.19	5.8	0.42	188.3	0.38	4041.3	1632.2	0.6
3	8.2	0.24	6.4	0.36	186.9	0.39	8317	3345.9	0.3
1	11.8	0.19	6.12	0.3	191.6	0.48	11352.9	5678.4	0.1

Supplementary references

1. Krinichnyi, V. I., Yudanova, E. I., Denisov, N. N., Konkin, A. A., Ritter, U., Bogatyrenko, V. R. & Konkin, A. L. Light-induced electron paramagnetic resonance study of charge transport in fullerene and nonfullerene PBDB-T-based solar cells. *J. Phys. Chem. C* **125**, 12224–12240 (2021).

2. He, S., Luo, X., Liu, X., Li, Y. L. & Wu, K. F. Visible-to-ultraviolet upconversion efficiency above 10% sensitized by quantum-confined perovskite nanocrystals. *J. Phys. Chem. Lett.* **10**, 5036–5040 (2019).

3. Lin, X., Chen, Z., Han, Y., Nie, C., Xia, P., He, S., Li, J. & Wu, K. ZnSe/ZnS core/shell quantum dots as triplet sensitizers toward visible-to-ultraviolet B photon upconversion. *ACS Energy Lett.* **7**, 914–919 (2022).
