# Supplementary material for: High-yield visible-to-ultraviolet upconversion by ZnSe quantum dots with dynamic triplet transfer
Source: Nat Commun. 2026 Jul 17;17:8777. doi: 10.1038/s41467-026-75613-5 (PMC13493803; doi:10.1038/s41467-026-75613-5)
Supplement: Supplementary file 2 [file 41467_2026_75613_MOESM2_ESM.pdf]

nature portfolio

Peer Review File

High Yield Visible-to-Ultraviolet Upconversion by ZnSe Quantum Dots with Dynamic Triplet Transfer

Corresponding Author: Professor Zihao Xu

This file contains all reviewer reports in order by version, followed by all author rebuttals in order by version.

Version 0:

Reviewer comments:

Reviewer #1

(Remarks to the Author)
In this paper, the highest photon upconversion quantum yield (UCQY) for QD-based light absorbers to date is reported using ZnSe quantum dots to absorb visible light to produce UV light via triplet-triplet annihilation. The authors establish that the highest UCQY occurs when the mean free path between the emitter molecules is 3 nm, matching the diameter of the QDs, indicating that these acceptors are poised to receive the triplet exciton from the QD. This steady-state UCQY measurements are accompanied by careful transient absorption spectroscopy outlining the change in rate. This high upconversion QY is all the more remarkable given that no transmitter ligand is needed. In other systems, this couldn't be achieved because the solubility of the annihilator is lower. Finally, using 400nm excitation, the authors show that photopolymerization of an acrylate can occur.

The following changes should be made to improve clarity
-Scheme 1b should be drawn to scale with accurate energy levels.
-Fig. 1c should have the excitation wavelength for upconversion listed. Maybe this reviewer missed it, but this information is not provided in the main paper or the supporting information. Is it the same as the excitation in Fig. 1a at 370nm? If so, this is not visible excitation

Reviewer #2

(Remarks to the Author)
The authors report in this manuscript on ligand-free quantum dot-based triplet-triplet annihilation upconversion, observing high upconversion efficiencies and characterizing the excited-state dynamics using transient spectroscopy. Although the presented results are intriguing, the study remains at a relatively preliminary stage overall. The most critical issue is that the authors fail to provide sufficient experimental data and in-depth discussion to elucidate why efficient upconversion can be achieved without the need for surface ligands. This issue constitutes the core mechanistic question of the study, yet the manuscript lacks an effective response to it.

When evaluating upconversion performance, upconversion efficiency and anti-Stokes shift are equally important. However, in this manuscript, an excitation wavelength of 380 nm is used, while the upconversion emission peak is located at approximately 350 nm. Although this wavelength relationship could technically be described as an upconversion process, it deviates considerably from the generally accepted understanding within the photon upconversion community. Similar phenomena can be observed in many organic small-molecule dyes simply by exciting the tail of their absorption band. Therefore, the upconversion performance of this system requires further validation and improvement.

Considering the potential for dynamic weak interactions between heteroatoms in PPO and the quantum dot surface in solution, such interactions have been demonstrated in some studies to facilitate triplet energy transfer. For instance, Chemical Science has reported that weak interactions between sulfur atoms in annihilators and PbSe quantum dots can promote triplet exciton transfer. However, this manuscript does not investigate such possible mechanisms nor provide direct evidence supporting the dominant role of this pathway.

In summary, given the lack of solid data supporting the key mechanistic interpretation and the controversies surrounding the

performance characteristics, I believe this manuscript is not yet suitable for publication.

Reviewer #3

(Remarks to the Author)
This work demonstrates a transmitter-free TTA-UC system using ZnSe QDs and PPO emitter, achieving a record 24% quantum yield. It reveals how emitter concentration modulates triplet energy transfer and TTA efficiency, establishing a critical design principle for optimizing QD-sensitized upconversion. The findings are significant and mechanistically insightful. Some questions should be answered before reconsideration:

1. The novelty of this work is the achievement of the record upconversion QY. A careful analysis of the spectroscopic data is needed. However, I don’t see any details about how upconversion QY is defined and calculated.

2. How do traps of ZnSe QD contribute to triplet transfer and upconversion? Trap emission disappears after adding PPO from Figure 1a. Do traps mediate triplet transfer from QDs to PPO that makes the record upconversion efficiency?

3. What are peaks at 4.8 and 4.9 ppm in ZnSe QD assigned to in the proton NMR in Figure S2?

Version 1:

Reviewer comments:

Reviewer #1

(Remarks to the Author)
The authors have addressed all reviewer comments adequately. This paper should be accepted.

Reviewer #2

(Remarks to the Author)
The authors have completed the revisions and recommend publication in Nature Communications.

Reviewer #3

(Remarks to the Author)
All concerns have been well responded, and details have been provided for quantum yield calculation. Recommend accepting the manuscript as is.

Open Access This Peer Review File is licensed under a Creative Commons Attribution 4.0 International License, which permits use, sharing, adaptation, distribution and reproduction in any medium or format, as long as you give appropriate

credit to the original author(s) and the source, provide a link to the Creative Commons license, and indicate if changes were made.
In cases where reviewers are anonymous, credit should be given to 'Anonymous Referee' and the source.
The images or other third party material in this Peer Review File are included in the article's Creative Commons license, unless indicated otherwise in a credit line to the material. If material is not included in the article's Creative Commons license and your intended use is not permitted by statutory regulation or exceeds the permitted use, you will need to obtain permission directly from the copyright holder.
To view a copy of this license, visit https://creativecommons.org/licenses/by/4.0/

Reviewer comments in black, our responses in blue and revisions in red

----

Reviewer 1:

In this paper, the highest photon upconversion quantum yield (UCQY) for QD-based light absorbers to date is reported using ZnSe quantum dots to absorb visible light to produce UV light via triplet-triplet annihilation. The authors establish that the highest UCQY occurs when the mean free path between the emitter molecules is 3 nm, matching the diameter of the QDs, indicating that these acceptors are poised to receive the triplet exciton from the QD. This steady-state UCQY measurements are accompanied by careful transient absorption spectroscopy outlining the change in rate. This high upconversion QY is all the more remarkable given that no transmitter ligand is needed. In other systems, this couldn't be achieved because the solubility of the annihilator is lower. Finally, using 400nm excitation, the authors show that photopolymerization of an acrylate can occur.

1. Scheme 1b should be drawn to scale with accurate energy levels.

Response:

Thank you for the valuable suggestion. Based on the photoluminescence (PL) peak of ZnSe quantum dots (QDs) at 397 nm, the exciton energy level is calculated to be 3.1 eV. The conduction band edge and valence band edge of ZnSe is ~-2.5 V and -5.6 V vs. vacuum (***ACS Energy Lett.*** 2022, 7, 914). The singlet and triplet exciton energy of PPO are 3.6 eV and 2.3 eV, the HOMO and LUMO are at -2.3 V and -5.9 V vs. vacuum (***J. Phys. Chem. Lett.*** 2019, 10, 5036. ***J. Phys. Chem. C*** 2021, 125, 12224). The energy level diagram has been redrawn with accurate scale. The revised figures are provided in Figure R1 and R2. Scheme 1b in the main text is revised and Supplementary Figure 1 is added in Supplementary Information.

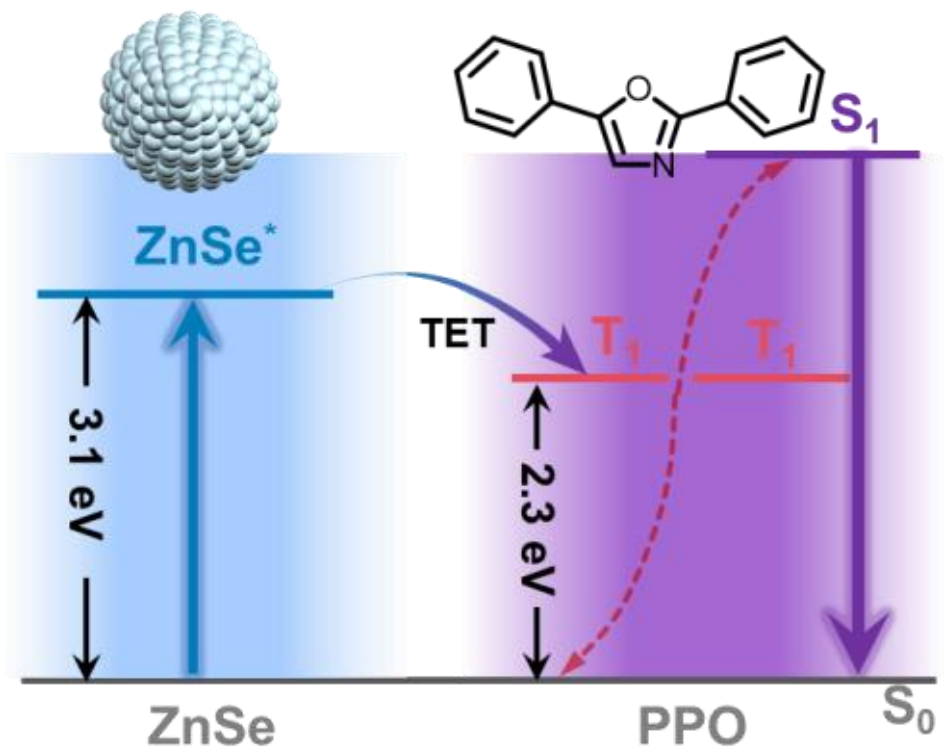

Figure R1. Transmitter-free ZnSe QD-sensitized visible-to-UV upconversion system developed in this work, consisting ZnSe QDs as the photosensitizer and PPO as the annihilator. In this configuration, the ZnSe exciton (3.1 eV) directly transfers energy to the PPO triplet state (2.3 eV) without intermediate acceptors. Note: ZnSe exciton energy is calculated from the PL peak, PPO triplet energy is obtained from previous literature.

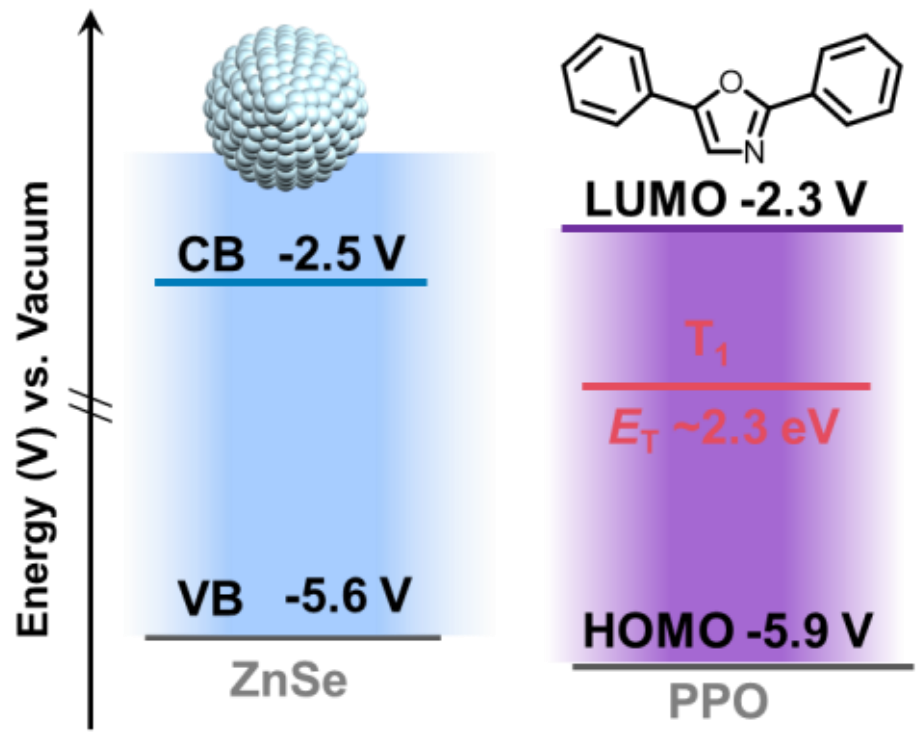

Figure R2. Conduction and valence band edge of ZnSe QD and HOMO, LUMO of PPO (***ACS Energy Lett.*** 2022, 7, 914; ***J. Phys. Chem. Lett.*** 2019, 10, 5036; ***J. Phys. Chem. C*** 2021, 125, 12224).

Revision:

We modified Scheme 1b and its caption on page 4. Figure R2 is added as Supplementary Figure 1 in Supplementary Information.

2. Fig. 1c should have the excitation wavelength for upconversion listed. Maybe this reviewer missed it, but this information is not provided in the main paper or the supporting information. Is it the same as the excitation in Fig. 1a at 370nm? If so, this is not visible excitation.

Response:

Thank you for the insightful comment, we didn't label the excitation wavelength in Figure 1 caption. The excitation wavelength used for the upconversion experiment in Fig. 1c is 380 nm, and this information has now been added in the figure caption. We agree that 380 nm is in the UV region. This was done for the efficiency of the upconversion.

To address your concern, we investigated upconversion performance of the ZnSe PPO hybrids at longer excitation wavelength using 405 nm excitation. As shown in Figure R3, UCQY under 405 nm excitation is 16.9%, lower than that under 380 nm excitation but the anti-Stokes is increased from ~230 meV to ~431 meV.

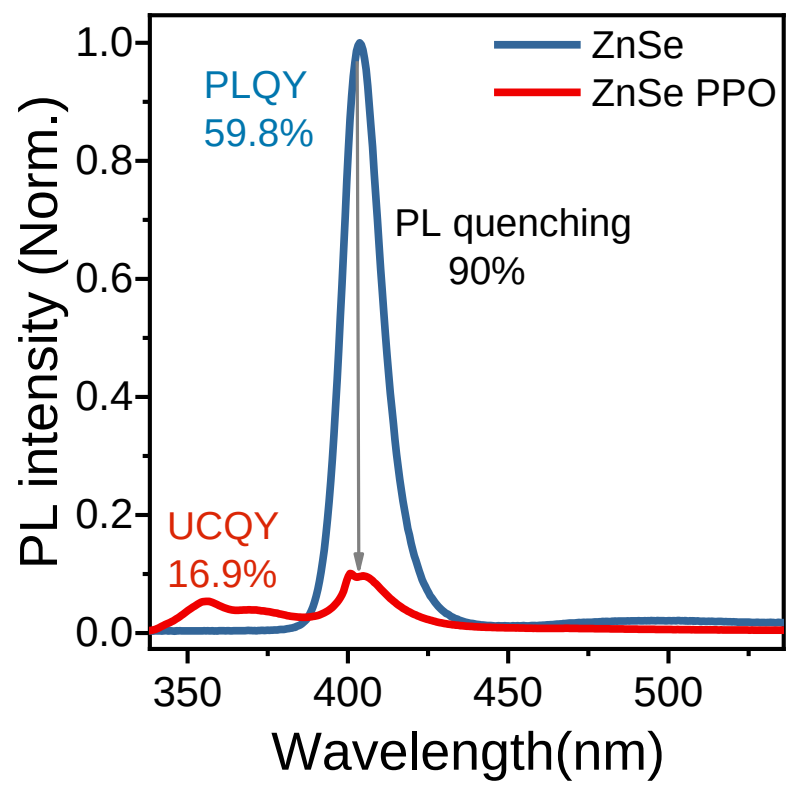

Figure R3. PL spectra of ZnSe and ZnSe PPO under 405 nm excitation.

Revision:

The caption of Fig. 1c has been revised to include the excitation wavelength. Supplementary Fig. 3 has been added in the SI to show upconversion under 405 nm excitation. We added the description in the main text on page 6.

"Considering the 380 nm excitation lies near the UV region, we further investigated TTA-UC performance of the ZnSe PPO hybrids under 405 nm excitation (Supplementary Fig. 3). As it shows, UCQY under 405 nm excitation is 16.9%, lower than that under 380 nm excitation but the anti-Stokes is increased from ~230 meV to ~431 meV. Considering the inhomogeneity of ZnSe QD (2.9 ± 0.9 nm), this reduction of anti-Stokes shift can be attributed to the increased QD size, which leads to a lower degree of exciton wavefunction delocalization beyond the QD surface, thereby decreasing the electronic coupling with molecular orbitals. In addition, the reduced energetic driving force further limits the efficiency of the Dexter-type TET process."

Reviewer 2:

The authors report in this manuscript on ligand-free quantum dot-based triplet-triplet annihilation upconversion, observing high upconversion efficiencies and characterizing the excited-state dynamics using transient spectroscopy. Although the presented results are intriguing, the study remains at a relatively preliminary stage overall. The most critical issue is that the authors fail to provide sufficient experimental data and in-depth discussion to elucidate why efficient upconversion can be achieved without the need for surface ligands. This issue constitutes the core mechanistic question of the study, yet the manuscript lacks an effective response to it.

Response:

Thank you. We agree that understanding why efficient upconversion can be achieved in the absence of surface ligands is critical to this study. In the response, we provided additional data and discussion to deepen the understanding of the upconversion mechanism.

1. When evaluating upconversion performance, upconversion efficiency and anti-Stokes shift are equally important. However, in this manuscript, an excitation wavelength of 380 nm is used, while the upconversion emission peak is located at approximately 350 nm. Although this wavelength relationship could technically be described as an upconversion process, it deviates considerably from the generally accepted understanding within the photon upconversion community. Similar phenomena can be observed in many organic small-molecule dyes simply by exciting the tail of their absorption band. Therefore, the upconversion performance of this system requires further validation and improvement.

Response:

Thank you for the constructive comment. We agree that UCQY and anti-Stokes shift are critical parameters for evaluating photon upconversion systems. When the excitation wavelength is 380 nm and the upconvert emission is 355 nm, the anti-Stokes

shift of 230 meV is modest. To address this issue, we further investigated TTA-UC performance under 405 nm excitation. As shown in Figure R3, the anti-Stokes shift reaches 431 meV with the UCQY of 16.9% under 405 nm excitation. Considering the inhomogeneity of ZnSe QD (2.9 ± 0.9 nm), this reduction of anti-Stokes shift can be attributed to the increased size of actually excited QD, which leads to a lower degree of exciton wavefunction delocalization beyond the QD surface, thereby decreasing the electronic coupling with molecular orbitals. In addition, the reduced energetic driving force further limits the efficiency of the Dexter-type TET process.

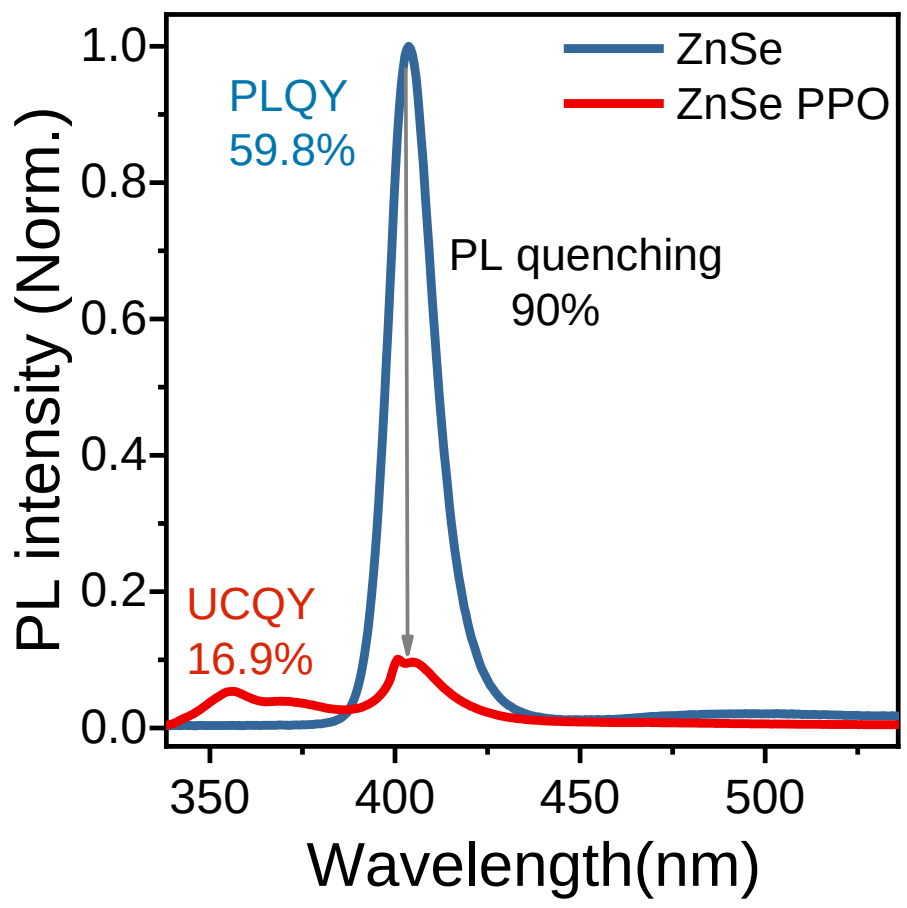

Figure R3. PL spectra of ZnSe and ZnSe PPO under 405 nm excitation.

We would like to show that the 350 nm emission does not originate from thermally assisted hot-band absorption but from a two-photon TTA kinetic process. We plot the excitation power versus emission intensity of the upconversion system to show that the UV emission originates from the two triplet upconversion process. The UC intensity vs. excitation power in Figure R4 shows that the slope is 2 before the excitation power threshold and changes to 1 after the threshold. The upconversion excitation power threshold indicates the process requires two triplets, which supports the triplet-triplet annihilation mechanism. Control experiment of direct exciting PPO at 30 mM under 380 nm excitation shows no emission at 350 nm, thus excluding the emission from thermally assisted hot-band absorption (***Adv. Sci.*** 2022, 9, 2202885). As Figure R5 shows, under both 1.63 W cm^{-2} and 5.85 W cm^{-2} excitation power densities, no emission was detectable around 355 nm.

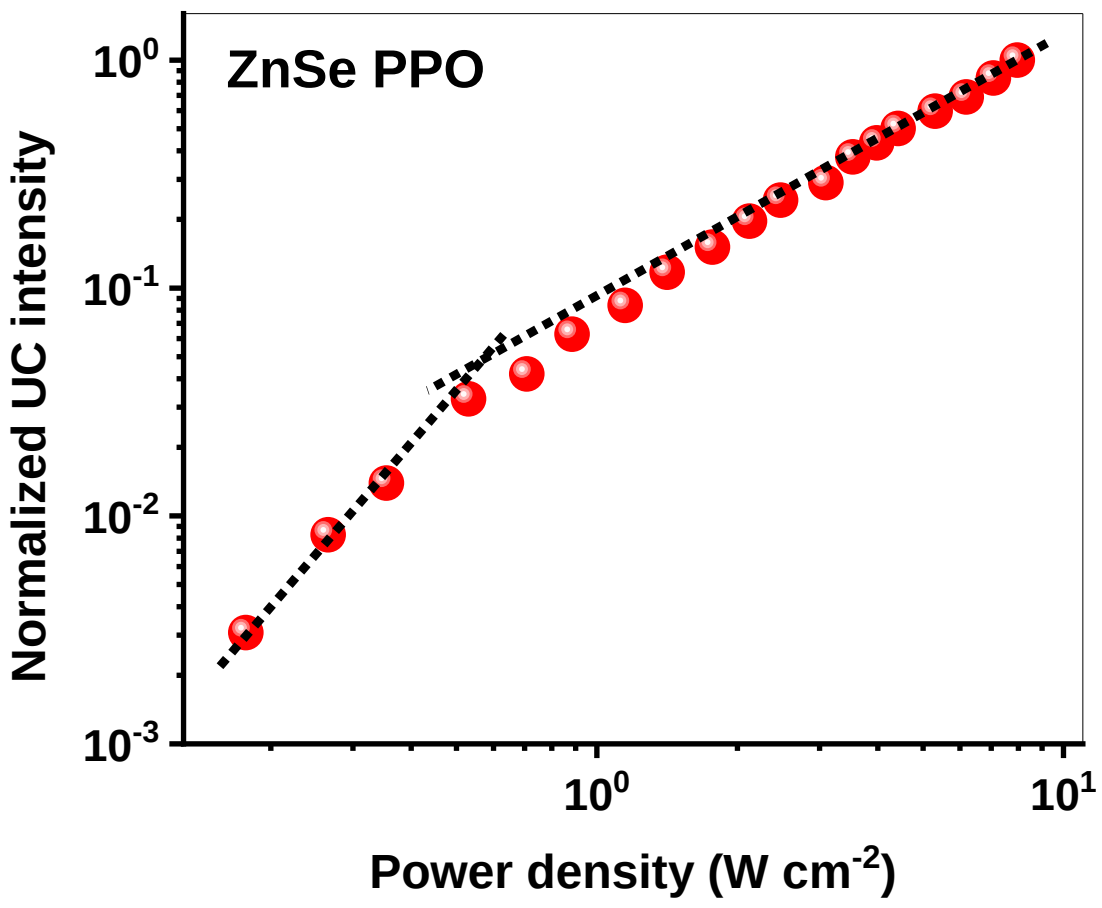

Figure R4. Excitation power threshold of the upconversion by ZnSe PPO.

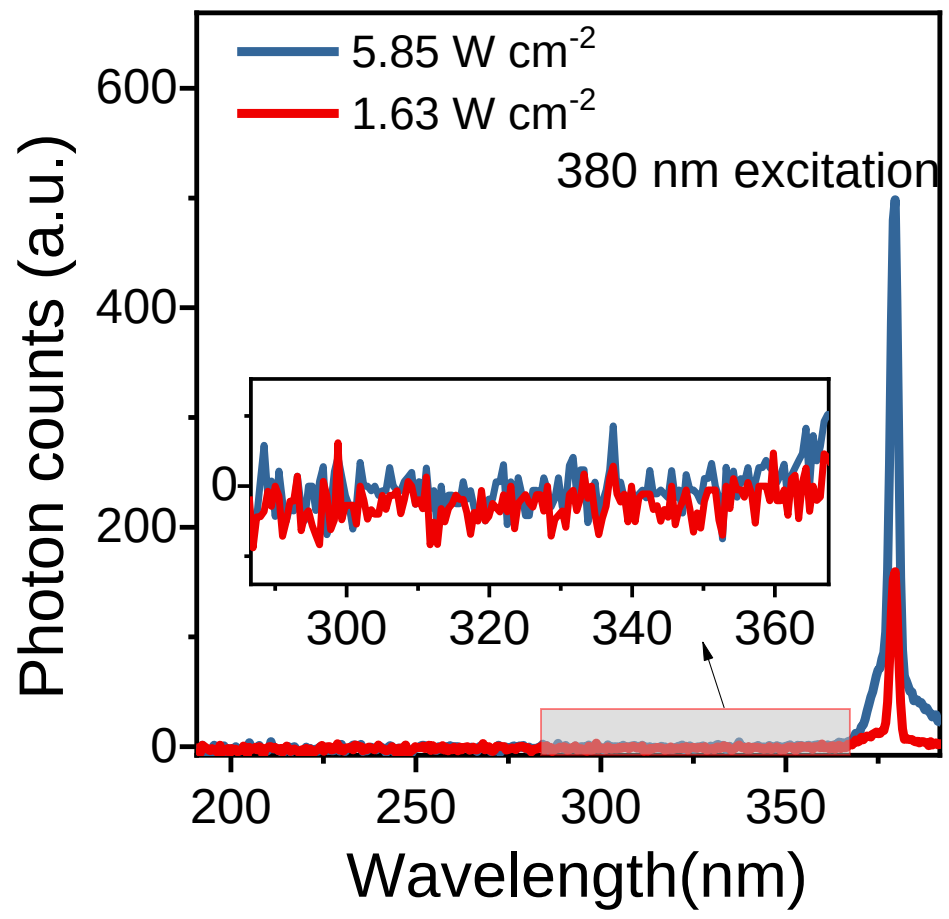

Figure R5. PL spectra of 30 mM PPO under 380 nm excitation with 1.63 W cm^{-2} and 5.85 W cm^{-2} power densities.

Revision:

We added the Figure R3 and R5 as Supplementary Fig. 3 and Supplementary Fig. 4 in SI, and added the discussion on page 6 in the main text:

"Considering the 380 nm excitation lies near the UV region, we further investigated TTA-UC performance of the ZnSe PPO hybrids under 405 nm excitation (Supplementary Fig. 3). As it shows, UCQY under 405 nm excitation is 16.9%, lower than that under 380 nm excitation but the anti-Stokes is increased from ~230 meV to ~431 meV. Considering the inhomogeneity of ZnSe QD (2.9 ± 0.9 nm), this reduction of anti-Stokes shift can be attributed to the increased size of actually excited QD, which leads to a lower degree of exciton wavefunction delocalization beyond the QD surface, thereby decreasing the electronic coupling with molecular orbitals. In addition, the reduced energetic driving force further limits the efficiency of the Dexter-type TET process. Control experiment of direct exciting PPO at 30 mM under 380 nm excitation shows no emission at 350 nm, thus excluding the emission from thermally assisted hot-band absorption (***Adv. Sci.*** 2022, 9, 2202885). As Supplementary Figure 4 shows, under both 1.63 W cm^{-2} and 5.85 W cm^{-2} excitation power densities, no emission was detectable around 355 nm."

2. Considering the potential for dynamic weak interactions between heteroatoms in PPO and the quantum dot surface in solution, such interactions have been demonstrated in some studies to facilitate triplet energy transfer. For instance, Chemical Science has reported that weak interactions between sulfur atoms in annihilators and PbSe quantum dots can promote triplet exciton transfer. However, this manuscript does not investigate such possible mechanisms nor provide direct evidence supporting the dominant role of this pathway.

Response:

Thank you for the insightful comment. This comment urges us to consider role of the dynamic weak interaction in this non-attaching binary system. After additional experiments, we have a new understanding of the physical picture. In summary, the dynamic weak interaction is more profound at low PPO concentration, and the direct TET via tunneling mechanism dominates at high PPO concentration. The turning point is the optimal PPO concentration at 30 mM, at which concentration the combination of both mechanism benefits both the TET and TTA efficiency. Below are the detailed experimental results:

1. Damping coefficient of triplet energy transfer rate shows a conversion from tunneling mechanism to dynamic interaction at the optimal PPO concentration. As the PPO concentration decreases, the triplet energy transfer rate decays exponentially with a damping coefficient β. Our previous study shows that if the acceptor has a dynamic interaction with QD, the acceptor could penetrate the capping ligand shell and accept the QD triplet at close proximity, then the acceptor leaves QD for the TTA process. As a result, the triplet energy transfer rate could show a shallow dependence with the capping ligand shell thickness (***J. Phys. Chem. Lett.*** 2022, 13, 3002), where k_{TET} shows a damping coefficient of 0.27 nm^{-1} with changing ligands length. In this work, k_{TET} in Figure 3 has a smaller β=0.09 nm^{-1} when the mean free path exceeds 3.8 nm (PPO concentration=30 mM), indicates a similar dynamic mechanism. A larger β=0.97 nm^{-1} is observed when the mean free path between PPO decreases below 3.8 nm, which can be attributed to the tunneling mechanism that TET can occur without relying on the PPO diffusion. At high PPO concentration exceeding 30 mM, the tunneling mechanism dominates the TET process, while the dynamic mechanism dominates at low PPO concentration. Actually, in our previous study (***J. Phys. Chem. Lett.*** 2022, 13, 3002), transmitter-free CdSe DPA TTA-UC was also realized despite visible emission (430 nm) and lower UCQY (6.9 %), wherein DPA doesn’t contain any sulfur or nitrogen atoms that may be involved in weak interactions with QDs.

2. As noted by the reviewer, ***Chem. Sci.*** 2019, 10, 4750 demonstrates that weak coordination interaction between S atom in thiophene-containing annihilator and metal atom on the PbS QD surface can promote molecular adsorption effect, enhancing electronic coupling and facilitating TET. The evidence for the coordination interaction includes a measurable blue shift (of 2 nm) of the QD excitonic absorption peak. In the present work, PPO contains an oxazole heterocycle, and the N atom could in principle interact with surface Zn atom of the ZnSe QD. The excitonic absorption peak of ZnSe QDs remains unchanged upon addition of PPO as shown in Figure R6. ^{1}H NMR of the

ligand on QD shows broadening effect and shift if absorbed on QD surface as reported in reference (***ACS Nano*** 2025, 19, 27246; ***New J. Chem.*** 2019, 43, 8452). Under identical concentration for PPO with QD (in cyclohexane-d12) and free PPO (in cyclohexane-d12), the ^{1}H NMR spectra show negligible changes in the chemical shifts of PPO protons (Figure R7), indicating that PPO molecules remain predominantly in a freely diffusing state rather than being coordinated to the QD surface.

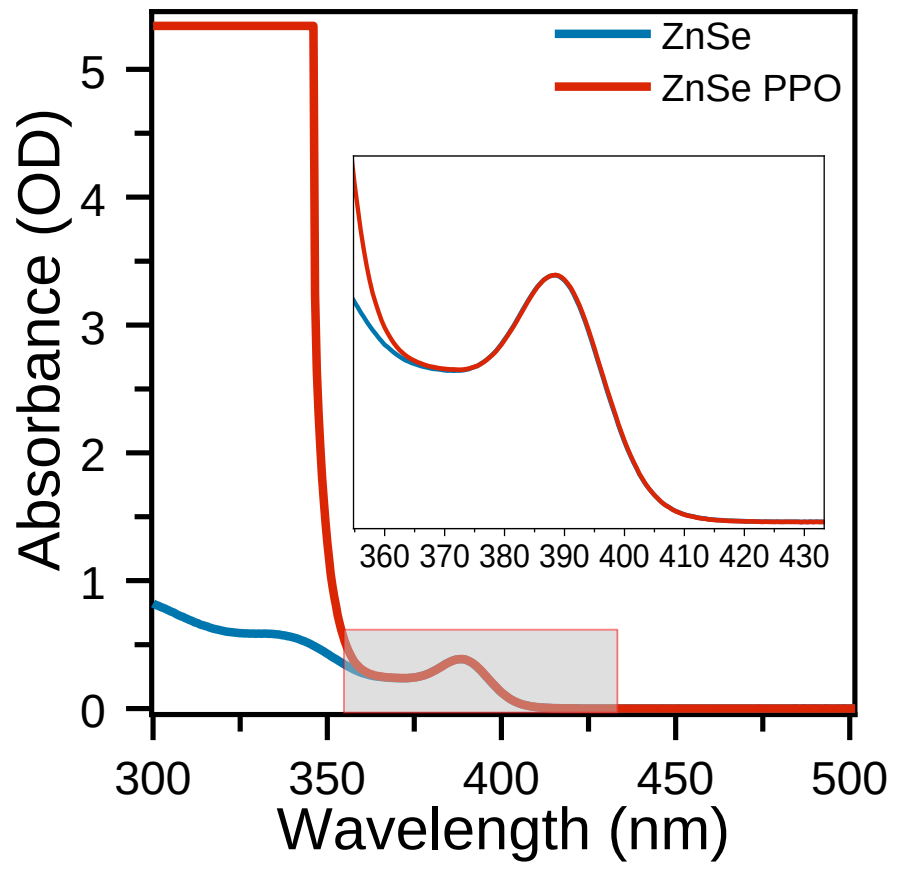

Figure R6. Absorption spectra of ZnSe (blue) and ZnSe PPO (red). The inset shows a magnified view of the region around the first exciton absorption peak.

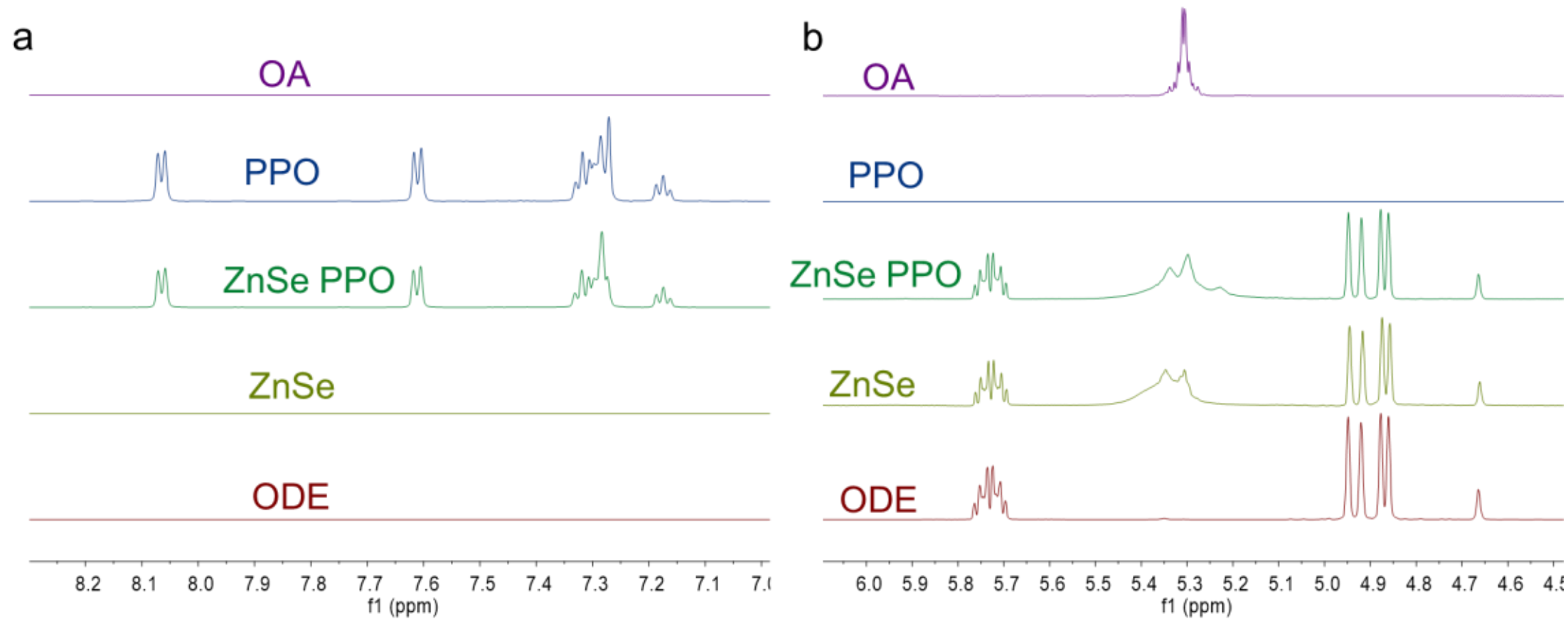

Figure R7. ^{1}H NMR spectra (in cyclohexane-d12) of OA (violet), PPO (blue) ZnSe PPO (green), ZnSe (yellow) and ODE (red). The PPO concentration is 30 mM in the PPO and ZnSe PPO. The QD concentration is 10 mg/mL in the ZnSe PPO and ZnSe.

We have revised the manuscript to include our new understanding and the new experimental results.

Revision:

We added Supplementary Fig. 5 and Supplementary Fig. 6, and added the description in the manuscript on page 11 and 12.

"In the reference (***Chem. Sci.*** 2019, 10, 4750), it has also been reported that the weak interactions between sulfur atoms in annihilators and PbS enabling dynamic attachment and detachment promote triplet exciton transfer. However, unlike the

measurable blue shift of the QD excitonic absorption peak in the reference (***Chem. Sci.*** 2019, 10, 4750), no measurable shift in the excitonic absorption peak of ZnSe QDs is observed upon addition of PPO (Supplementary Fig. 5). The NMR of the PPO on QD shows that the PPO molecules remain predominantly in a freely diffusing state rather than being coordinated to the QD surface (Supplementary Fig. 6)."

"By controlling the molecular distance, we believe that the transformation from a tunneling mechanism to a dynamic mechanism occurs with the mean free path increasing over 3.8 nm. The highest UCQY is obtained exactly at the turning point. Specifically, TA analysis on k_{TET} in Figure 3 reveals much larger β (0.97 nm^{-1}) below 3.8 nm mean free path, which can be attributed to the tunneling mechanism that TET can occur without relying on diffusion. On the contrary, when the mean free path exceeds 3.8 nm, the much smaller β (0.09 nm^{-1}) indicates a similar dynamic mechanism where the acceptor diffuses close to the QD (***J. Phys. Chem. Lett.*** 2022, 13, 3002). Consequently, at high PPO concentrations exceeding 30 mM, the tunneling mechanism dominates the TET process, while the dynamic mechanism dominates at low PPO concentrations."

3. In summary, given the lack of solid data supporting the key mechanistic interpretation and the controversies surrounding the performance characteristics, I believe this manuscript is not yet suitable for publication.

Response:

Thank you for evaluating the scientific rigor of this manuscript and for highlighting important concerns regarding mechanistic interpretation and performance characteristics. We fully appreciate the reviewer's emphasis on the need for solid experimental evidence and clear mechanistic understanding. In this response, we have substantially revised the manuscript by incorporating additional experimental results and expanded discussion to strengthen the validity of the proposed mechanism and clarify the origin of the observed upconversion behavior.

Specifically, multiple lines of evidence have been provided to support efficient TTA-UC in the absence of surface ligands. (1) Transient absorption spectroscopy reveals accelerated exciton decay kinetics of the QDs upon addition of PPO, accompanied by spectral signatures consistent with $^3PPO^*$ formation, directly confirming the occurrence of TET. (2) No measurable shift in the excitonic absorption peak is observed after addition of PPO, and ^1H NMR spectra show negligible chemical shift changes, indicating that PPO does not strongly coordinate to the QD surface through heteroatom binding. (3) Systematic variation of PPO concentration significantly alters the average intermolecular diffusion distance, and enhanced TET efficiency is observed at shorter diffusion lengths. These results collectively support the mechanistic picture proposed in this work: in solution-phase systems, increasing annihilator concentration reduces the mean free path, thereby increasing the probability of effective QD–molecule encounters and enhancing short-range Dexter-type electronic exchange interactions required for TET.

We emphasize that the primary contribution of this work is the identification of a previously underexplored design strategy for QD-sensitized TTA-UC systems, namely

achieving efficient TET without relying on polycyclic aromatic surface ligands. Instead of extending excited-state lifetime through ligand engineering, the present work demonstrates that modulation of molecular diffusion kinetics can provide an alternative route to enable efficient Dexter-type energy transfer. Based on the additional experimental evidence and expanded mechanistic discussion provided in the revised manuscript, we believe that the key scientific conclusions are better supported. We sincerely hope that the revised version meets your standard, we truly appreciate the constructive comments that improves the clarity and rigor of the manuscript.

Reviewer 3:

This work demonstrates a transmitter-free TTA-UC system using ZnSe QDs and PPO emitter, achieving a record 24% quantum yield. It reveals how emitter concentration modulates triplet energy transfer and TTA efficiency, establishing a critical design principle for optimizing QD-sensitized upconversion. The findings are significant and mechanistically insightful.

1. The novelty of this work is the achievement of the record upconversion QY. A careful analysis of the spectroscopic data is needed. However, I don't see any details about how upconversion QY is defined and calculated.

Response:

Thank you. In the revised manuscript, we clarified the definition of UCQY, detailed the calibration procedure, and provided additional information regarding error control and reproducibility.

In this work, UCQY is defined as the ratio between the number of emitted upconverted photons and the number of absorbed excitation photons. Following the standard convention in the TTA-UC field, the UCQY is expressed as:

$$\Phi_{UC} = 2\Phi_{C307}\frac{(1-10^{-A_{C307}})}{(1-10^{-A_S})}\frac{F_S}{F_{C307}}\frac{n_S^2}{n_{C307}^2} \qquad \text{(R1)}$$

Where A_i is the absorption (optical density) at the excitation wavelength, F_i is the integrated photon number and n_i is the refractive index of the solvent. Subscripts C307 and S are coumarin 307 and sample, respectively. It should be noted that, since the upconversion PL of PPO overlaps to some degree with the emission of ZnSe QDs, F_s is calculated by normalizing the pure PPO PL to the corresponding characteristic peak for the PL of QDs PPO hybrids.

The spectral response function of the detection system was calibrated using a photon-number-based method following a previously reported procedure (***J. Phys. Chem. Lett.*** 2019, 10, 5036). Specifically, a femtosecond optical parametric amplifier (fs-OPA) was used to generate monochromatic excitation at different wavelengths. By measuring the laser power at each wavelength, the corresponding photon flux was calculated, allowing the construction of a calibration curve correlating detector signal intensity with absolute photon number.

To verify the accuracy of the calibration curve, two well-established fluorescent

dyes, PPO and anthracene (ANT), were employed as cross-calibration standards. Based on literature values, PPO exhibits a fluorescence quantum yield of approximately 85% in cyclohexane (***J. Mol. Struct.*** 1999, 508, 19). Using the calibrated spectral response function, we determined the fluorescence quantum yield of anthracene to be 26% (Figure R8), which agrees well with widely accepted literature values (***J. Chem. Phys.*** 1971, 54, 1). This consistency confirms the reliability of the instrument calibration.

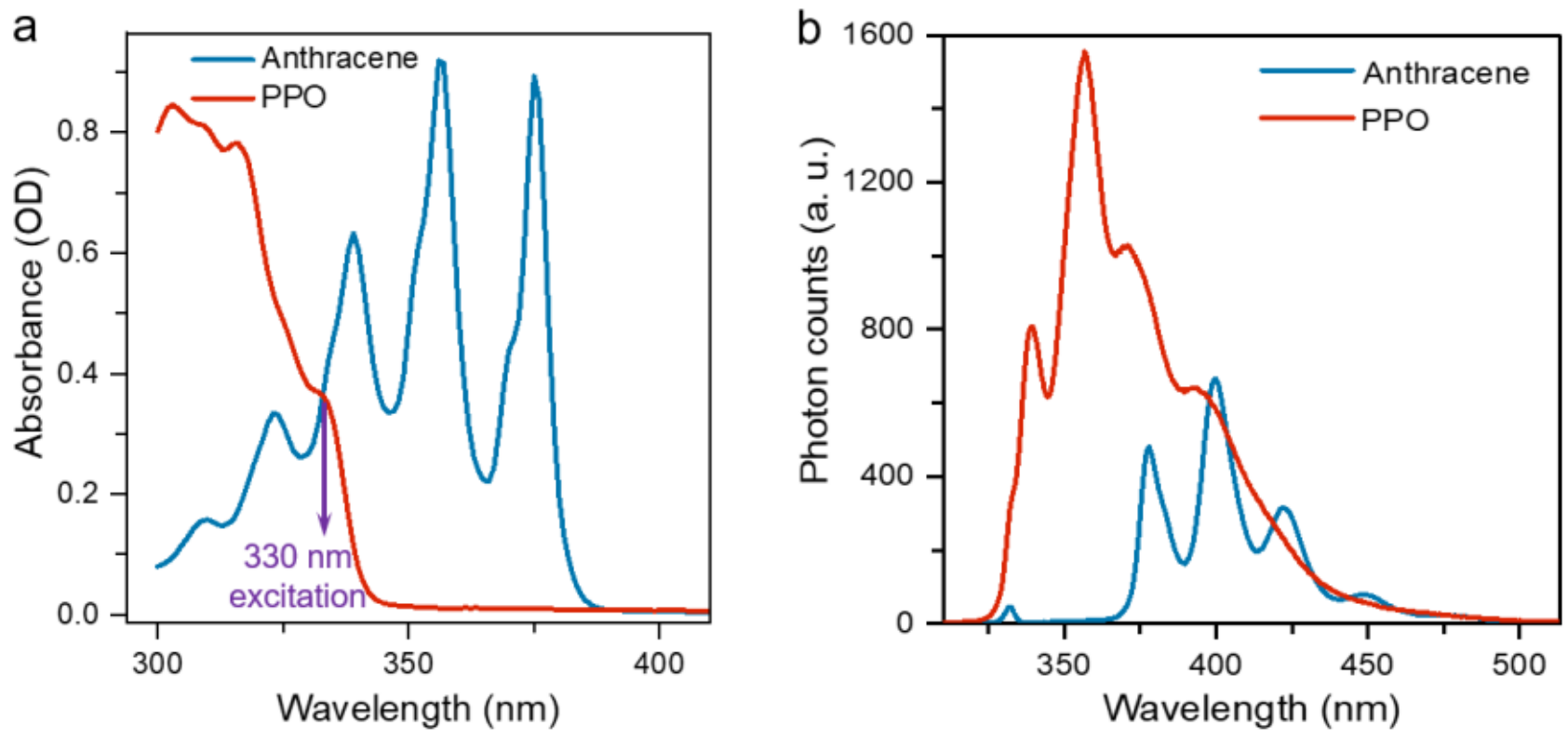

Figure R8. (a) Absorption spectra of anthracene (blue) and PPO (red). Note: the absorbance at 330 nm for these two molecules is tuned to be identical. (b) PL spectra of anthracene (blue) and PPO (red) under the 330 nm laser excitation. Note: the excitation source is the fs pulse produced by OPA.

Through the combination of absolute spectral calibration and cross-validation with standard fluorophores, we believe the reported UCQY value is reliable.

Revision:

We added Supplementary Fig. 7 and Supplementary Note 1 in SI.

"TTA-UC measurements

UCQY is defined as the ratio between the number of emitted upconverted photons and the number of absorbed excitation photons. Following the standard convention in the TTA-UC field, the UCQY is expressed as:

$$\Phi_{UC} = 2\Phi_{C307}\frac{(1-10^{-A_{C307}})}{(1-10^{-A_S})}\frac{F_S}{F_{C307}}\frac{n_S^2}{n_{C307}^2} \quad \text{Supplementary Equation (1)}$$

Where A_i is the absorption (optical density) at the excitation wavelength, F_i is the integrated photon number and n_i is the refractive index of the solvent. Subscripts C307 and S are coumarin 307 and sample, respectively. It should be noted that, since the upconversion PL of PPO overlaps to some degree with the emission of ZnSe QDs, F_s is calculated by normalizing the pure PPO PL to the corresponding characteristic peak for the PL of QDs PPO hybrids.

The spectral response function of the detection system was calibrated using a photon-number-based method following a previously reported procedure. Specifically, a femtosecond optical parametric amplifier (fs-OPA) was used to generate monochromatic excitation at different wavelengths. By measuring the laser power at each wavelength, the corresponding photon flux was calculated, allowing the construction of a calibration curve correlating detector signal intensity with absolute

photon number.

To verify the accuracy of the calibration curve, two well-established fluorescent dyes, PPO and anthracene, were employed as cross-calibration standards. Based on literature values, PPO exhibits a fluorescence quantum yield of approximately 85% in cyclohexane. Using the calibrated spectral response function, we determined the fluorescence quantum yield of anthracene to be 26% (Supplementary Fig. 7), which agrees well with widely accepted literature values. This consistency confirms the reliability of the instrument calibration."

2. How do traps of ZnSe QD contribute to triplet transfer and upconversion? Trap emission disappears after adding PPO from Figure 1a. Do traps mediate triplet transfer from QDs to PPO that makes the record upconversion efficiency?

Response:

Thank you. As shown in Figure 1a, the ZnSe QDs exhibit a trap-related emission centered at ~485 nm, which disappears upon addition of PPO. This observation indicates that the presence of PPO modifies the excited-state relaxation pathways of the QDs. We agree that trap states may participate in TET processes, for example via transfer from trap states to the $^3PPO^*$. However, based on steady-state PL, transient spectroscopy, and quantitative efficiency considerations, we conclude that trap-mediated transfer is unlikely to be the dominant pathway responsible for the high UCQY.

First, considering the relative contribution of different emissive channels, the PL of ZnSe is dominated by band-edge exciton emission at 397 nm, while the trap emission contributes only a minor fraction of the total PL intensity, with a PLQY of ~8.3%. Even assuming an idealized scenario in which triplet energy transfer from trap states to PPO occurs with 100% efficiency, the limited population of trap-state excitations would not be sufficient to account for the observed UCQY of 24%.

Second, upon addition of PPO, the band-edge exciton emission is strongly quenched (~95%), indicating efficient electronic interaction between the QD exciton and PPO molecules. Transient absorption spectroscopy further provides direct evidence for efficient TET, as reflected by accelerated decay of the QD exciton signal accompanied by the appearance of characteristic absorption features attributable to the $^3PPO^*$. These results clearly support efficient TET occurring directly from the band-edge exciton state.

As a result, while trap states may contribute to excited-state kinetics to some extent, the dominant mechanism responsible for the high TTA-UC efficiency is efficient Dexter-type TET from the band-edge exciton state of the QDs to the $^3PPO^*$.

Revision:

We added description in the manuscript on page 8 and 9.

"We also note that the emission from the ZnSe trap state is strongly quenched upon addition of PPO. It suggests that trap states could participate in TET, for example via

transfer to $^3PPO^*$. However, steady-state PL, transient spectroscopy, and quantitative efficiency analysis indicate that trap-mediated transfer is unlikely to dominate the observed high UCQY. Firstly, the PL of ZnSe is primarily governed by band-edge exciton emission at 397 nm, while trap emission represents only a minor fraction of the total PL intensity (PLQY ~8.3%). Even assuming unity TET efficiency from trap states, their limited population would be insufficient to explain the observed UCQY of 24%. Secondly, addition of PPO leads to strong quenching of the band-edge exciton emission (~95%), indicating efficient electronic coupling between QDs and PPO. TA measurements further confirm efficient TET, evidenced by accelerated decay of the exciton signal together with the emergence of characteristic $^3PPO^*$ absorption features. These results clearly support direct TET from the band-edge exciton as the dominant pathway."

3. What are peaks at 4.8 and 4.9 ppm in ZnSe QD assigned to in the proton NMR in Figure S2?

Response:

We thank the reviewer for this insightful question. The ^1H NMR signals observed at 4.8 and 4.9 ppm in Figure S2 are assigned to the olefinic protons of 1-octadecene (ODE). Similar chemical shifts for the vinyl protons of ODE have been reported in the literature (***ACS Nano*** 2016, 10, 2071), typically appearing in the 4.8–5.0 ppm region.

To further confirm this assignment, we measured the ^1H NMR spectrum of pure ODE in the same solvent system (cyclohexane-d12), as shown in Figure R7. The olefinic proton signals of ODE match well with the peaks observed at 4.8 and 4.9 ppm in Figure R7, confirming that these resonances originate from residual ODE rather than PPO or other species potentially interacting with the QD surface. ODE is commonly used as a high-boiling solvent in quantum dot synthesis, and small residual amounts after purification are frequently observed in colloidal nanocrystal systems. Since ODE does not contain functional groups capable of strongly coordinating to the ZnSe surface, its presence does not affect the TET mechanism discussed in this work.

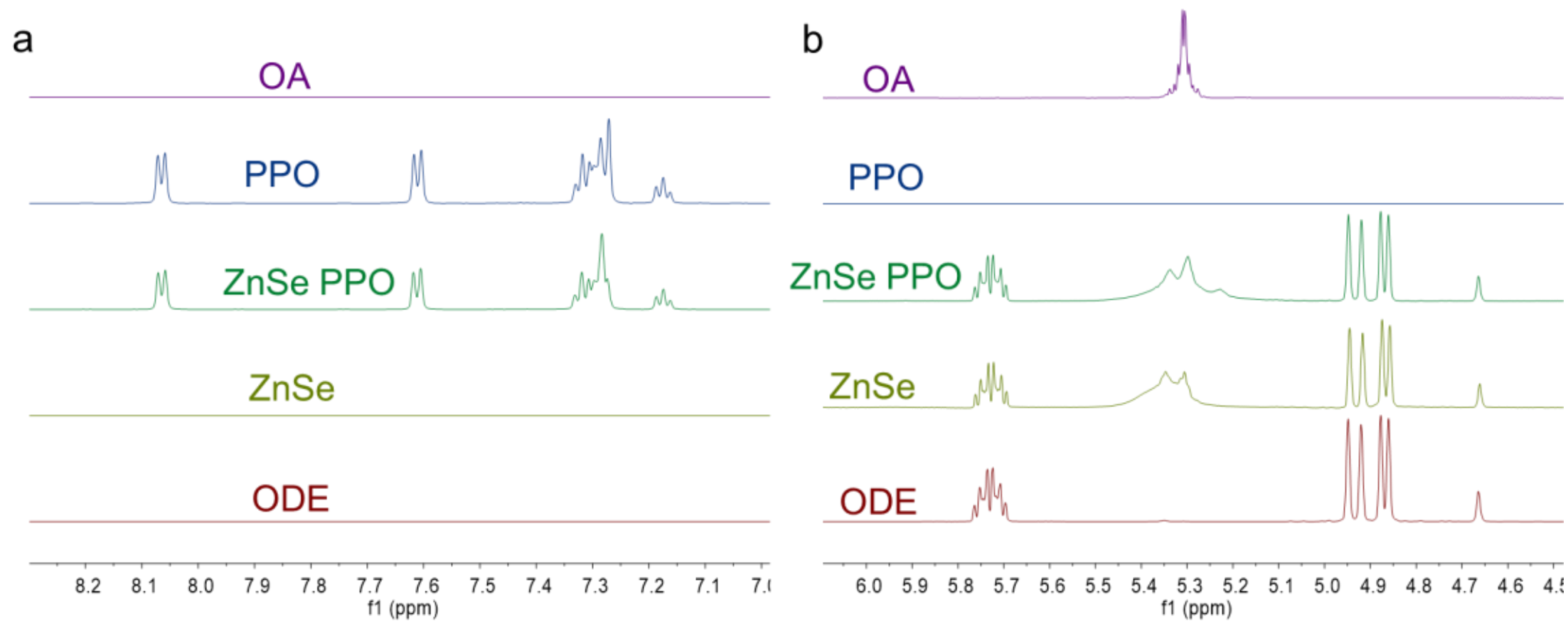

Figure R7. ^1H NMR spectra (in cyclohexane-d12) of OA (violet), PPO (blue) ZnSe PPO (green), ZnSe (yellow) and ODE (red). The PPO concentration is 30 mM in the PPO and ZnSe PPO. The QD concentration is 10 mg/mL in the ZnSe PPO and ZnSe.

Revision:

We modified Figure S2 as new Supplementary Fig. 6 in Supplementary Information.
